# Emergent mechanics of actomyosin drive punctuated contractions and shape network morphology in the cell cortex

**DOI:** 10.1371/journal.pcbi.1006344

**Published:** 2018-09-17

**Authors:** Callie J. Miller, Demetrius Harris, Robert Weaver, G. Bard Ermentrout, Lance A. Davidson

**Affiliations:** 1 Bioengineering, University of Pittsburgh, Pittsburgh, PA, United States of America; 2 Bioengineering, Pennsylvania State University, State College, PA, United States of America; 3 Mathematics, University of Pittsburgh, Pittsburgh, PA, United States of America; Purdue University, UNITED STATES

## Abstract

Filamentous actin (F-actin) and non-muscle myosin II motors drive cell motility and cell shape changes that guide large scale tissue movements during embryonic morphogenesis. To gain a better understanding of the role of actomyosin *in vivo*, we have developed a two-dimensional (2D) computational model to study emergent phenomena of dynamic unbranched actomyosin arrays in the cell cortex. These phenomena include actomyosin punctuated contractions, or "actin asters" that form within quiescent F-actin networks. Punctuated contractions involve both formation of high intensity aster-like structures and disassembly of those same structures. Our 2D model allows us to explore the kinematics of filament polarity sorting, segregation of motors, and morphology of F-actin arrays that emerge as the model structure and biophysical properties are varied. Our model demonstrates the complex, emergent feedback between filament reorganization and motor transport that generate as well as disassemble actin asters. Since intracellular actomyosin dynamics are thought to be controlled by localization of scaffold proteins that bind F-actin or their myosin motors we also apply our 2D model to recapitulate *in vitro* studies that have revealed complex patterns of actomyosin that assemble from patterning filaments and motor complexes with microcontact printing. Although we use a minimal representation of filament, motor, and cross-linker biophysics, our model establishes a framework for investigating the role of other actin binding proteins, how they might alter actomyosin dynamics, and makes predictions that can be tested experimentally within live cells as well as within *in vitro* models.

## Author summary

Recent genetic and mechanical studies of embryonic development reveal a critical role for intracellular scaffolds in generating the shape of the embryo and constructing internal organs. In this paper we developed computer simulations of these scaffolds, composed of filamentous actin (F-actin), a rod-like protein polymer, and mini-thick filaments, composed of non-muscle myosin II, forming a two headed spring-like complex of motor proteins that can walk on, and remodel F-actin networks. Using simulations of these dynamic interactions, we can carry out virtual experiments where we change the physics and chemistry of F-actin polymers, their associated myosin motors, and cross-linkers and observe the changes in scaffolds that emerge. For example, by modulating the motor stiffness of the myosin motors in our model we can observe the formation or loss of large aster-like structures. Such fine-grained control over the physical properties of motors or filaments within simulations are not currently possible with biological experiments, even where mutant proteins or small molecule inhibitors can be targeted to specific sites on filaments or motors. Our approach reflects a growing adoption of simulation methods to investigate microscopic features that shape actomyosin arrays and the mesoscale effects of molecular scale processes. We expect predictions from these models will drive more refined experimental approaches to expose the many roles of actomyosin in development.

## Introduction

Dynamic actomyosin networks play a critical role in development by providing motive forces for cell shape change and morphogenesis, and by establishing tissue mechanical properties [1–3]. For instance, actomyosin can form a contractile actin purse-string, a rope-like structure of bundled F-actin spanning multiple cells at the margin of the lateral epidermis that contracts and contributes to dorsal closure in *Drosophila* [4–6]. Contractile actomyosin networks in the medioapical domain of epithelial cells can also drive cell shape change leading to bending of epithelial sheets during gastrulation in *Drosophila* [7]. In addition to regulating force or stress production actomyosin is responsible for establishing the mechanical properties of the embryo that resist stress and guide tissue deformation. For instance, actomyosin controls much of the viscoelastic properties of *Xenopus* during gastrulation and neurulation as dorsal axial tissues converge and extend [8–10]. On the cellular scale, the biomechanical function of actomyosin is a direct target of many signaling pathways that pattern cell identities and behaviors in the embryo. For instance, Wnt-signaling during mediolateral cell intercalation appears to control force production and stiffness by regulating F-actin polymerization and myosin II contractility [11, 12]. Given the importance of actomyosin networks and their relevance to most, if not all, morphogenetic processes during development, we know a great deal about their composition and molecular-scale processes. By contrast, surprisingly little is known about the mechanisms that coordinate the large-scale spatial and temporal dynamics of actomyosin network assembly and contraction during morphogenesis.

**Fig 1:**
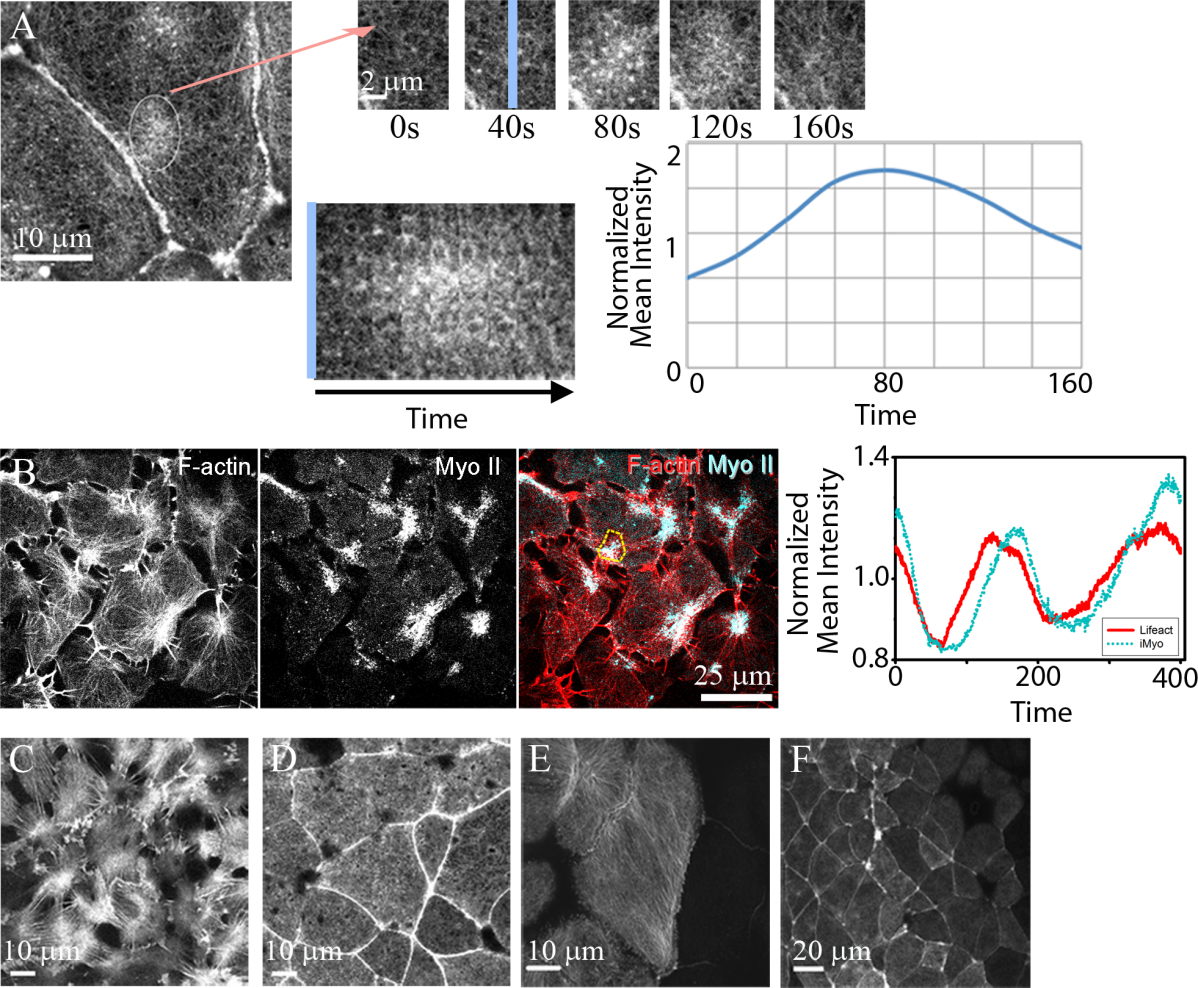
Kinetics and diverse morphology of actomyosin contractions in *Xenopus laevis* embryos. (A) Punctuated F-actin contractions, or "actin-asters" are observed the apical cortex of *Xenopus* neural epithelial cells during gastrulation. F-actin is seen across the apical surface and cell-cell junctions but is transiently enriched during a contraction (circle). A series of frames from a time-lapse sequence (arrow) reveals rapid accumulation and dissipation of F-actin in the apical cortex. A kymograph of the contraction (across blue line) shows the changing intensity of F-actin and quantified as changes in normalized intensity (I_contraction_/I_cell_) over length of the time lapse. Normalized intensity is based on identifying a region of interest (ROI) and tracking the intensity with that ROI over time. (B) Frames from a time-lapse series where F-actin (mChe-life-act) and mini-thick filaments of active myosin II (MyoII; mNeon-sf9, [22]) can be tracked in the basal cell cortex. Temporal analysis reveals that myosin co-localizes with F-actin asters but lags both accumulation and dissociation profiles of F-actin. (C) Similar spatial and temporal patterns of F-actin are evident on the basal surface of a *Xenopus* animal cap explant cultured on fibronectin coated glass, (D) the apical surface of an animal cap explant cultured against a clean glass surface, (E) the apical surface of the blastopore lip (confocal image provided by Joseph Shawky and Rafey Feroze, personal communication, March 2014), and (F) across a broad field of cells of the neural epithelium (confocal image provided by Deepthi Vijayraghavan, personal communication, June 2017).

Live imaging of fluorescently tagged F-actin and myosin regulatory light chain have revealed that actomyosin networks in the cell cortex are very dynamic, forming transient structures that turn-over or remodel in minutes [13–16]. Unbranched F-actin networks within the cell cortex are both less dense and less organized than F-actin in lamellipodia or circumapical junctions. Time-lapse sequences using fluorescent proteins conjugated to actin-binding domains from moesin or utrophin, or minimal synthetic actin-binding domains (e.g. life-act) reveal dynamic heterogeneous arrays of F-actin. Aster-like structures are often observed in these time-lapse sequences. Such asters form and persist for a few minutes before dissipating [11, 12, 17–20] (Fig 1A; S1 Video). The central cores of actin asters are enriched with active myosin II which appears to lag the assembly of the aster (Fig 1B; S2 Video). Asters are observed in a variety of morphogenetic events where they are strongly correlated with cell shape change [3, 21].

Actin asters can be seen on surfaces other than the apical cell cortex. F-actin networks in the basal cortex of the epithelium and basolateral cortex of mesenchymal cells in *Xenopus* embryos (Fig 1C) adopt less dense morphologies than those in the apical cell cortex (Fig 1D). A diverse range of F-actin morphologies, including asters in the apical cortex can be seen within different classes of epithelial cells even at the same stage of development: for instance, highly aligned F-actin in prospective epidermal cells bordering the blastopore lip (Fig 1E)[23], or high frequency actomyosin contractions in the neural epithelium as the neural folds form and neural tube closes (Fig 1F).

Complex actomyosin dynamics can also be studied *in vitro* as reconstituted gels (e.g. [24, 25]) or within microcontact printed arrays [26–28]. One advantage of reconstituted gel models is that their mechanical or rheological properties can be measured directly [29, 30] as the composition of the gel is changed, e.g. addition of purified actin cross-linking proteins [31]. Such studies reveal how factors that alter the morphology of stable actin networks correlate with changes in material properties. Microcontact printing has been used to create patterns of actin polymerizing protein (Nucleating Promoting Factor pWA), myosin motors (myosin VI or myosin II), or capping protein (CapZ) [26–28]. Once actin binding factors or motors are immobilized, or printed onto a glass substrate, purified G-actin and additional factors are added, and the evolution of the network is followed by time-lapse confocal or total internal reflection fluorescence microscopy. Actin dynamics that arise from printed patterns can serve as physical analogs of the native actin cortex, allowing more detailed correlation of cytoskeletal protein-protein interactions with mechanical properties and kinematics of actomyosin networks, and have provided many insights into *in vivo* actomyosin dynamics. Reconstituted *in vitro* systems can be viewed as physical analogs for cortical actin in the cell and complement theoretical computational analog models we describe next.

Complex dynamics of actomyosin arrays observed *in vivo* and *in vitro* have inspired theoretical and computational models seeking to connect the biophysical interactions of F-actin and myosin II motors to macroscopic phenomena [32]. Early biophysical models on the origin of muscle contraction forces were closely coupled to experiments; for instance, Huxley's model connected microscopic structural analysis of striated muscle [33] to dynamics and metabolism of contractility measured experimentally [34]. More recently efforts have sought to explain phenomenological events driven by less ordered arrays of actomyosin typically found in epithelial and mesenchymal cells. Such filament arrays have been the subject of theoretical and computational models investigating how interactions between actin filaments, myosin II motors and their regulators might drive emergence of ordered arrays or allow disordered arrays to generate or transmit force. Continuum models of actomyosin mechanics and bulk dynamics have emerged from studies of active polar polymers (e.g. [35]) to explain complex patterns of F-actin seen in cells (e.g. [36–42]). Such models often combine, or lump, specific parameters of actin and myosin biophysical interactions into bulk-stresses or modulus and have succeeded in representing mesoscopic behaviors of actomyosin seen in tissues, cells, and reconstituted actomyosin gels [25].

Live cell imaging of actomyosin dynamics have inspired development of microstructurally realistic computational models. One strategy to simulate complex actomyosin dynamics involves the adoption of agent based computational simulations similar to those used to study microtubule dynamics [43]. These approaches utilize agent-based microscopic models that represent individual actin filaments and proteins such as cross-linkers that interact to modulate filament connectivity. Individual mini-thick filaments of myosin II can be included to drive filament rearrangement (e.g. [44–51]). Each agent, or molecular component can interact with others through defined biochemical and biophysical processes to drive changes in the filament array. Our own microstructural actomyosin models have been motivated by these efforts and by the need to understand how actomyosin asters emerge within embryonic cells and how these structures may be coordinated and generate force during development [52].

Several groups have recently applied similar microscopic computational models to explore the complex morphologies that emerge from actomyosin interactions. Models have been used to understand the complex actin morphologies that emerge from *in vitro* experiments with recombinant proteins microcontact printed in patterns or confined in specific ring-like geometries [53, 54]. Other models have been constructed to understand the dual function of actomyosin arrays in both generating and transmitting forces to neighboring cells [50] or the role of actin polymerization and alignment during assembly and constriction of the cytokinetic furrow [42, 46]. Efforts similar to our own have also sought to understand remodeling of filament arrays by motors [51], the evolution of stress production and clustering of actin within the 2D cell cortex [55], and to explore the potential contribution of stress-induced actin filament severing [48] to actomyosin mechanics. Increasing computational requirements for these simulations led us to seek simplifications that would allow us to compare the importance of various protein-protein interactions and association rates that shape actomyosin arrays within the cell cortex and how cells might disassemble actin arrays or spatially control morphologies through localization of actin-binding proteins.

To understand how dynamic ordered arrays such as asters might emerge from disordered networks we developed a 2D "search, capture, remodel, and traffic" model that incorporates dynamic aspects of *in vivo* F-actin and myosin motor interactions. We find this model captures many of the observed behaviors of *in vitro* model system actomyosin. By including F-actin and myosin motor interactions and their effect on transporting myosin motors we have advanced beyond current computational and *in vitro* models, including our previous rotational model [47], to investigate the important biophysical interactions that shape actin asters. To extend microstructural models to study actomyosin networks confined to thin layers such as the embryonic cell cortex, we created a model of a two-dimensional array of actin filaments with myosin motors. Since there is a strong positive correlation between "material" elastic modulus and actin cross-linking in both reconstituted gels [56–60] and the cortical actin [61] we also chose to investigate the role of cross-linkers on filament-motor dynamics by implementing cross-linker agents and testing their effectiveness in shaping asters. Although changes in network morphology can be qualitatively correlated with specific perturbations, in this paper we investigate how actomyosin asters arise and may be shaped by changing conditions in the cell and discuss how programs of development and morphogenesis might control cortical actomyosin dynamics to drive and guide tissue movement.

## Results

### Actomyosin asters emerge from a sparse minimal network

**Fig 2:**
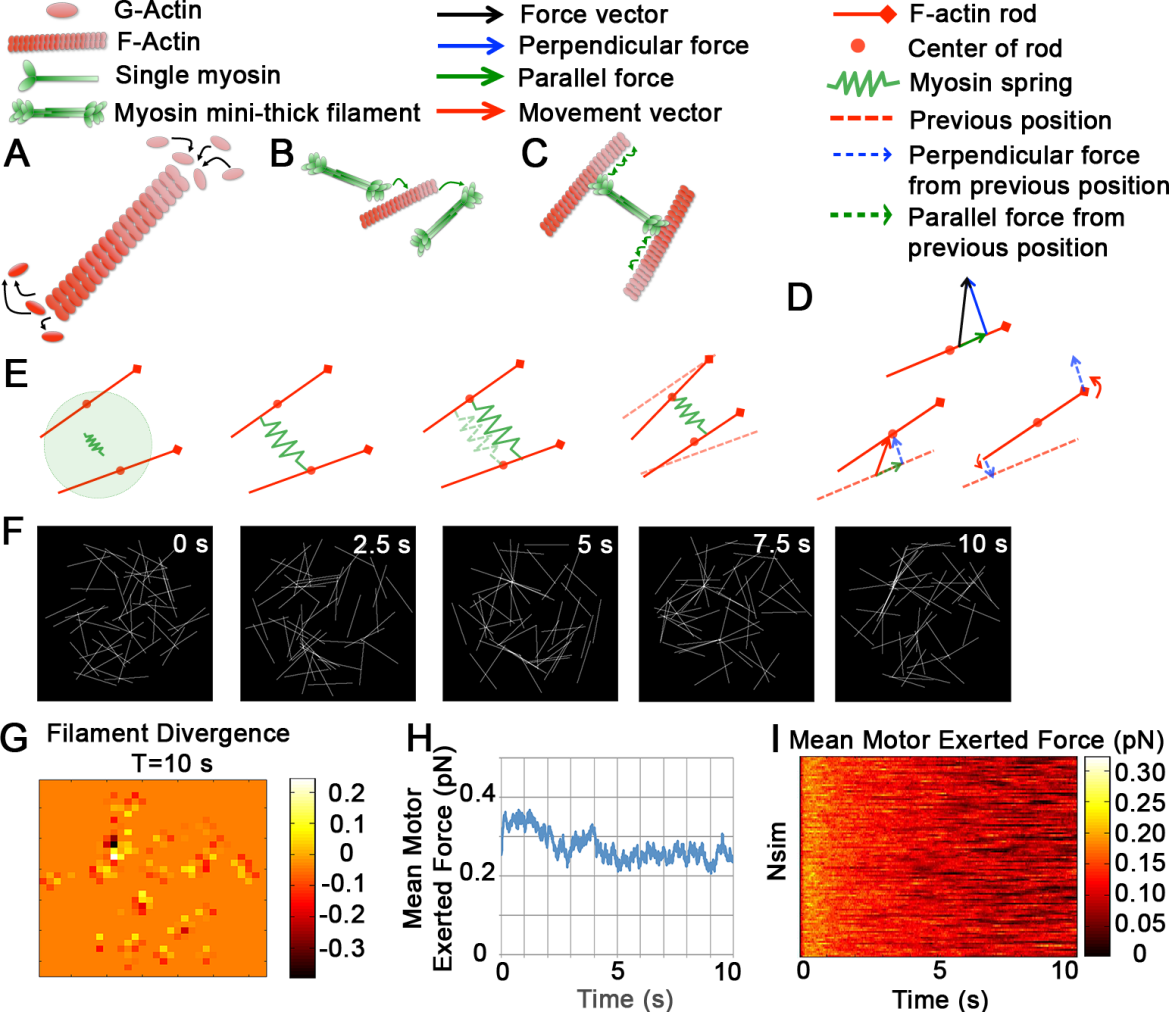
Dynamic model of filaments and motors in two dimensions. (A—E) Schematic depicting the biophysical interactions and dynamics of F-actin and myosin II motors. A) G-actin monomer extends the plus- or barbed-end of the actin filament through polymerization and disassembles the filament at the minus end. Our simulation does not explicitly implement polymerization or depolymerization dynamics but rather captures the impact of those processes through a single turn-over rate, i.e. one filament completely depolymerizes and a new one polymerizes at a new random location. (B) Individual non-muscle myosin II motors bundle together into a multi-headed mini thick filament and when activated bind to F-actin, or when inactivated detach from F-actin. Our simulation does not explicitly implement motor assembly, activation, or inactivation. Instead, our model motors represent non-muscle myosin II already bundled into activated myosin filaments. (C) Once bound, myosin filaments 'walk' to the plus-end of the F-actin. (D) Vector diagram of force generated by a single motor attached to a filament. The filament bound by the motor moves in 2D as vector forces at the attachment point are decomposed into parallel and perpendicular forces which are opposed by viscous drag. The perpendicular force (blue) leads to a perpendicular translation and torque applied to the ends drive filament rotation; forces applied parallel to the filament drive translational movement. (E) Schematic showing filament movements after a searching motor binds a pair of filaments: after the motor binds a pair of filaments each head domain moves at a fixed velocity toward the bound filament's plus-end. After one time step, the motor exerts a force couple on the filament pair, rotating and pulling the filaments together. Repeated applications of such directed forces result in polarity sorting of filaments that may have initially been anti-parallel. (F) A sparse network of 50 filaments (white) and 250 motors (not shown) illustrates filament movements over 1,000 time steps or 10 seconds. (G) Filament divergence, a measure of aster assembly, reveals a single aster at point of transition from low to high divergence. (H) The mean motor exerted force (blue; pN) over 1,000 time steps or 10 seconds. (I) The time-evolution of mean motor exerted force for 100 simulations, each initiated with randomly positioned filaments and motors.

To illustrate the biophysical processes incorporated in our actomyosin model, we first simulate a sparse network of 50 filaments and 250 motors (Fig 2A–2E). Each filament in our model represents a fixed length actin microfilament. Each motor represents a multiprotein complex of multiple non-muscle myosin II motors composed of heavy and light chains that self-assemble into a myosin filament [62] also referred to as a bipolar myosin II mini thick filament [52]. As motors bind and walk toward the plus- or barbed-end on pairs of filaments we observe polarity sorting and the co-emergence of a dense cluster of motors and filaments that we refer to as an “aster” (see Materials and Methods for model equations and implementation and S1 Table for model specific parameters; Fig 2F). Asters can be recognized by the divergence of their component filaments (see Materials and Methods for divergence calculations; Fig 2G; divergence indicates aster centers by sites where filament orientations reverse). Briefly, we assigned vectors originating from filament plus-ends and summed the orientation for box areas covering the domain ($\vec{V_{i,j}}=[V_{x_{i,j}},V_{y_{i,j}}]$). We then used a second order derivative approximation to determine the divergence in x (*divX*_*i*,*j*_) and y (*divY*_*i*,*j*_) for each box and summed these to determine the total divergence. As the aster forms, the motor-generated internal-network forces decrease to a steady state value (Fig 2H), and the network morphology reaches a dynamically stable form with filament plus-ends gathered at the aster center.

Each simulation begins with a disordered initial distribution of filaments and motors; to compare different conditions we carried out 100 simulations and compared the mean internal-network forces (Fig 2I). The steady state of force over 100 simulations is normally distributed (0.09 pN ± 36%, S1 Fig). The asters produced in our simulations, are formed as plus-ends of filaments pack together in the center with minus ends radiating outward. For the remainder of this study we present single simulations to represent behaviors observed over multiple repetitions from different initial configurations.

### Temporal evolution of an actomyosin aster

**Fig 3:**
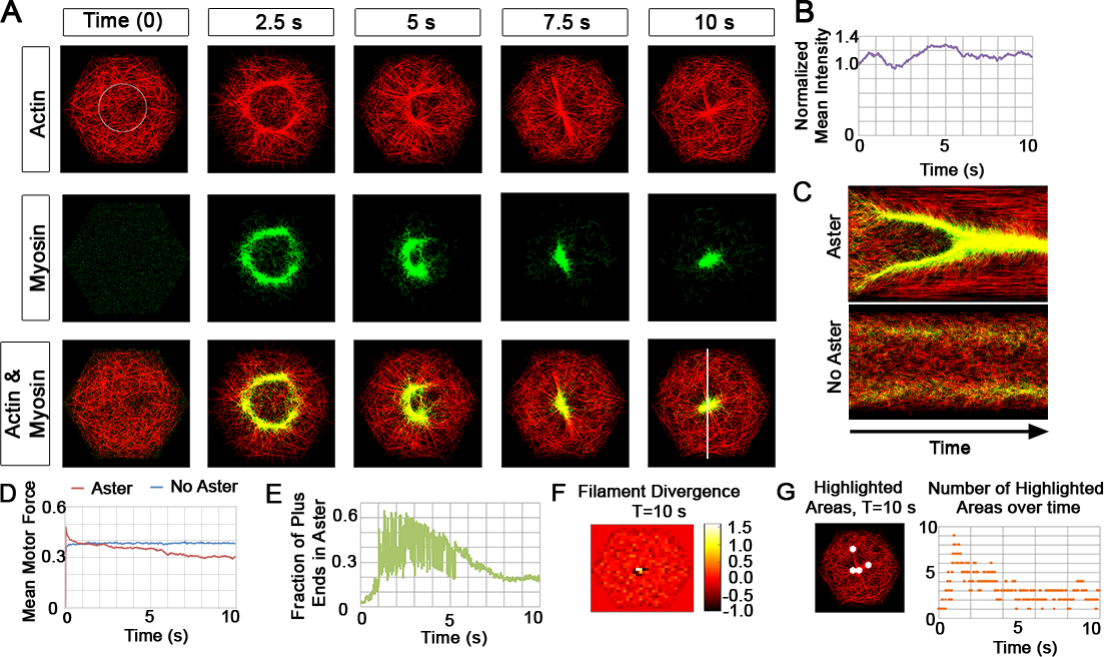
Actomyosin arrays remodel in simulated dense networks. (A) Sequence of images from a simulated time lapse of 1,000 actin filaments (red) and 5,000 myosin motors (green). (B) The normalized actin mean intensity (purple) within a region in A (circle at 0 s). The mean intensity is based on identifying the location of an aster within a region of interest (ROI; in this case a circle), and tracking the intensity in that ROI over time. (C) Kymograph at top show formation of an aster over 10 seconds (line in A at 10 s). Kymograph below shows a case where the filament turnover rate is high (p_2_, 5/s) and no asters are formed. (D) The mean motor exerted force quickly peaks and subsides in cases where asters form (pN, red) but remain constants for cases where no asters form (blue). We have compared the mean motor force for 10 simulations with the standard parameter set and observe that the profiles are similar (S2 Fig) (E) The 'connectedness' of filament plus-ends shown over the length of simulation. (F) The divergence of filaments at the conclusion of a simulation generating an aster; high divergence (yellow region in the center) indicates where filament polarity reverses. (G) Our coarse-grained image analysis technique shows highlighted hexagonal areas within the larger domain at 10 s (S4 Video). We quantified the number of the highlighted areas over time (orange). Additional calculations, including a comparison between the image analysis of filament intensity mappings versus divergence mappings are in the supplementary material (S3 Fig).

To understand dynamics of the model under more physiological conditions we increased filament and motor density to 1,000 filaments and 5,000 motors (Fig 3A; S3 Video). The denser actin network is reorganized by the myosin motors through an intermediate, isotropic ring structure (over the first 2.5 seconds; Fig 3A), which continues to contract into a stable aster (from 7.5 to 10 s; Fig 3A). To compare dynamic remodeling of model filament-motor arrays with *in vivo* data collected from time-lapse sequences we generated time-lapse sequences of models mimicking the linear additive fluorescence signal observed in confocal time-lapse sequences. Computed synthetic time-lapse sequences allowed us to analyze aster formation with image-based tools commonly applied to live-cell F-actin dynamics [11, 17, 63]. As an illustration, we track the normalized mean intensity of filaments within a fixed circular region of interest (ROI), to observe intensity increases over the first second as the network begins to form a ring structure, but drops after 2 seconds (Fig 3B) as the ring forms outside of the ROI. At this time, filaments are "swept" into the ring by motors and depleted in the center. Intensity at the center gradually increases as the ring of filaments and motors contract into the centralized aster from 2.5 to 7.5 s (Fig 3A, Fig 3B). The normalized mean intensity then maintains a high level reflecting compaction of the ring into the aster together with filament turnover since filaments are randomly depolymerized and relocated randomly to places within the hexagon, including regions outside of the ROI. A kymograph provides another method of quantifying the aster emergence (Fig 3C, upper panel) and also reveals movement of filaments into the aster similar to that seen in live-cell time-lapses of F-actin in *Xenopus* cells [11, 17]. For comparison, we include a kymograph from a simulation with high filament turnover (p_2_, 5 s^-1^) where asters do not assemble (Fig 3C, lower panel). Asters generated in our simulations are qualitatively similar to punctuated actomyosin contractions observed *in vivo* during epithelial morphogenetic movements such as *Drosophila* apical constriction [7], establishment of anterior-posterior polarity in *C*. *elegans* embryos [64, 65], and dorsal closure in *Drosophila* [66].

Internal-network forces and plus-end positions evolve in a pattern qualitatively similar to that observed in the sparse network. The internal network force (Fig 3D), and the filament divergence at the end of the simulation (Fig 3E and 3F) approach a steady-state similar to that reached by the sparse network (Fig 2H). Mean motor generated force at steady state in cases lacking aster formation is higher than the case when the aster is formed (Fig 3D). Furthermore, not all filaments are recruited into the aster (Fig 3E and 3F) due to the rapid transport and trapping of motors by the centrally concentrated plus-ends of the radial filament array. Polarized filaments in the aster direct all bound motors toward the aster's center (Fig 3A, myosin location in green). Motors are trapped by the aster core; as a motor moves to and falls off the filament plus-end in the core, it diffuses until it binds to another filament, however, at the core it only finds other plus-ends that all direct the motor into the aster center.

Our initial simulations demonstrate that aster formation is highly robust and that once assembled, an aster is a dynamically stable structure. In many cases actomyosin contractions observed in cells can be stable (e.g. asters in *Xenopus* at sites of bundled extracellular matrix, or in 'nodes' late in gastrulation) but often contractions are transient [11, 17]. To fully recapitulate the *in vivo* dynamics of punctuated actomyosin contractions, asters would need to dissipate after formation. Such pulsatile contractions are not an emergent feature in our model, rather, we suspect transition between a stable aster and its disassembly requires altered rules or conditions that control F-actin and myosin II function.

To understand the influence of biophysical properties of F-actin and myosin mini thick filaments on aster formation and stability we carried out a series of simulations varying these parameters. In most cases, simulations reach steady state within 1,000 time steps (10 s); longer simulations to 3,000 (30 s) or 6,000 (60 s) time steps were carried out on the remaining cases (see S2 Table and S1 Text for aster identification methods). Simulations were performed with each parameter allowed to vary through their physiological range (S1 Table).

### Actin filament dynamics regulate aster morphology

**Fig 4:**
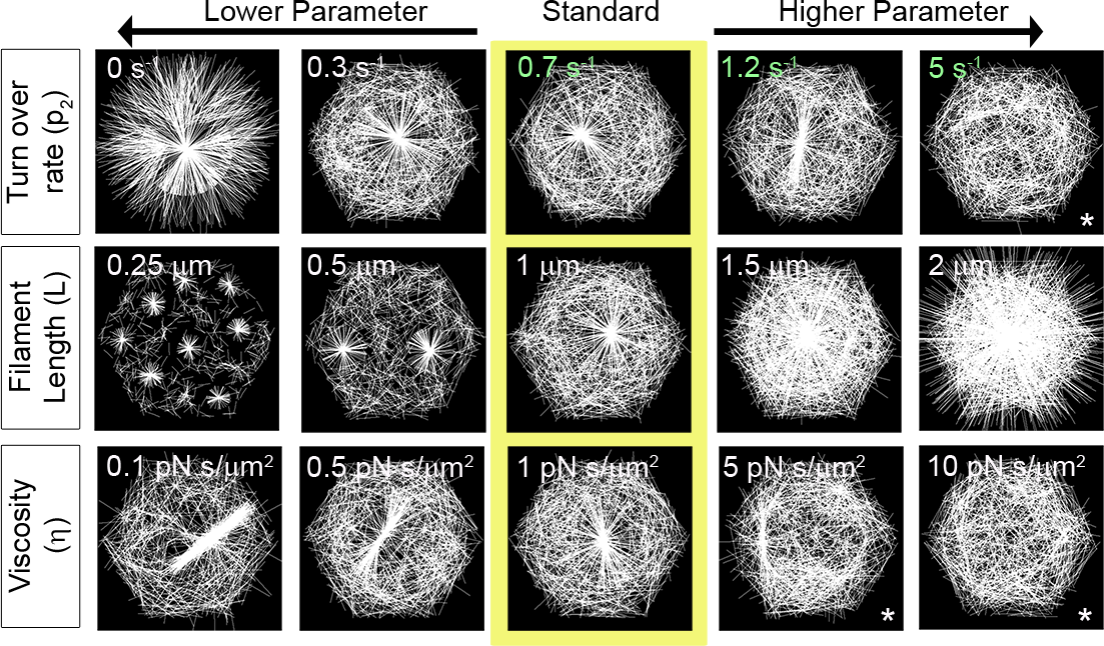
Diverse actomyosin arrays can be formed by discrete changes in filament dynamics. Steady state F-actin morphology after 10 s as F-actin biophysics is altered. Arrays from different simulations in the center column (yellow box) are formed by a standard set of parameters. Each row depicts arrays formed when a single parameter regulating F-actin is changed. Arrays formed from simulations are shown in each column and are ordered from lower (left) to higher parameter values (right). Only the parameter described by row and column were varied, the other parameter values were held at the standard value. See S1 Table for the details of parameters used here. We have included an asterisk (*) to note cases where an aster has not formed. To determine if an aster could form over longer times, we ran these simulations for a more extended duration and found only minor changes in the asters (S5 Fig).

In simulations where filament turn-over rates are zero (e.g. p_2_ set to 0), the majority of filaments quickly rearrange due to motor interactions, with plus-ends in the center, forming an aster (Fig 4 and S4 Fig). Rapid aster formation traps motors and limits their access and thus their interactions with non-aster filaments. Furthermore, the abundance of unbound motors is reduced outside the aster, limiting the domain contributing filaments to the forming aster. If we reduce the ability of the motors to stay attached to filaments, we slow the recruitment of filaments into the aster. As the turnover rate of filaments increases, asters can no longer form (p_2_, 5 s^-1^) since filaments are removed from nascent asters and are randomly redistributed. Once a filament turns over, any motor attachments are severed. Therefore, when the turnover rate of filaments is high, the time motors spend exerting forces on filaments to organize them into an aster is greatly reduced. Thus, aster formation can be slowed or inhibited by high rates of turnover or by reducing the length of time motors interact with filaments.

Changing filament length alters aster formation in a manner similar to alterations observed when filament density or turn-over is changed *in vivo*. Shorter filaments (L, 0.25 μm) result in formation of multiple small asters whereas longer filaments (L, 2 μm) create single, domain encompassing asters (Fig 4; S5 Video). Asters produced with short filaments are similar to actin networks observed *in vivo* after treatments with Cytochalasin D or Latrunculin B, which depolymerize actin to produce sparse actin networks [11]. Additionally, small asters have been observed to interact physically when filament length is reduced [67]. The formation of multiple asters can be attributed to reducing filament length as well as reducing the area searched by unbound motors.

One of the effects of motors on network remodeling can be understood in the mechanism of motor transport on filaments; bound motors move faster through the domain than by simple diffusion. When filaments are shorter, motors traverse shorter distances along each filament slowing the process of filament polarity sorting and assembly of filaments into asters. Motors cannot link short filaments from two different asters that would otherwise enable the merger of multiple smaller asters formed by larger filaments. Lastly, increased filament length leads to dense actin networks similar to those observed *in vivo* when F-actin is stabilized with Jasplakinolide [11].

Changing the dynamic viscosity, η, alters the rate that motor forces produce filament translation and rotational movement (5 pN s/μm^2^; the dynamic viscosity of water is 8.9x10^-4^ pN s/μm^2^). Simulations with a lower viscous cytoplasm (0.1 pN s/μm^2^) result in more bundling of filaments in the aster (Fig 4).

### Myosin motor dynamics regulate aster morphology

**Fig 5:**
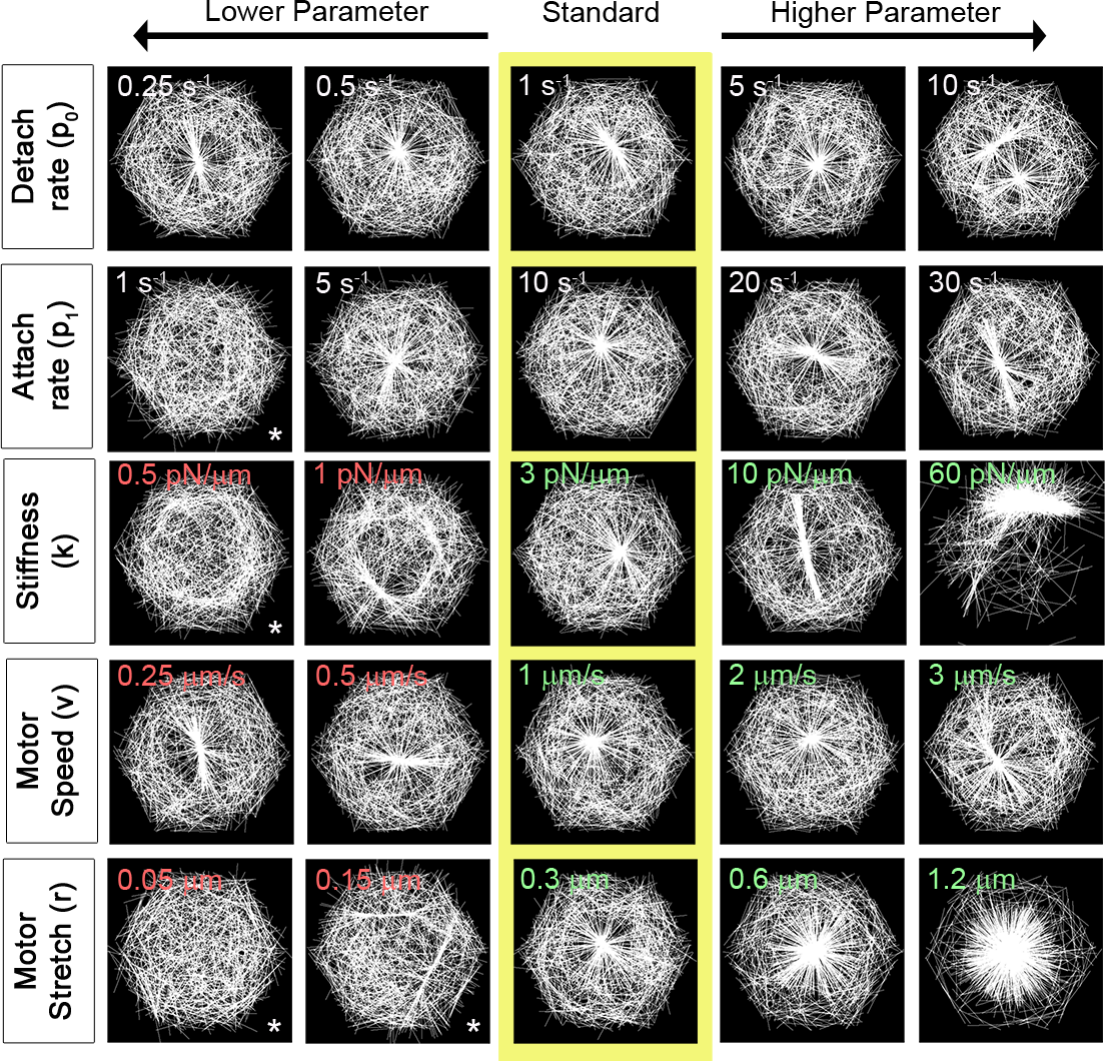
Diverse actomyosin arrays can be formed by discrete changes in motors dynamics. Steady state F-actin morphology after 10 time steps as myosin II biophysics is altered. The yellow box indicates the same set of standard parameters but random initial configuration. Each row depicts morphology generated by changes in a single parameter controlling myosin II motors, ordered from lower value on the left to higher value on the right. Each simulation starts with randomly oriented and positioned filaments and motors. Red numbers represent values outside of the physiological determined range, green numbers are values within physiological range, and white represent numbers whose ranges are best guess values. Rates of motor attachment (p_1_) and detachment (p_0_) are estimated. See S1 Table for additional parameters. We have included an asterisk (*) to note cases where an aster has not formed. To determine if an aster could form if given enough time, we ran these simulations for longer time and present results in S5 Fig.

With the exception of motor contractility, we see few changes in aster formation as we alter parameters that change motor interactions with filaments. Strongly increasing the rate of motor detachment (p_0_, 10 s^-1^; Fig 5) can contribute to the formation of multiple asters as motors exert short bursts of force onto filament pairs. Changing the rate of motor attachment only disrupts aster formation when the rate is low (p_1_, 1 s^-1^, Fig 5). Alternatively, motor contractility, or spring stiffness, can alter aster formation; extremely compliant motors, below physiologically measured values, are never able to rearrange filaments into a single, central aster, instead remaining more concentrated at the periphery or assembling multiple asters at the periphery (k, 0.5 or 1 pN/μm, Fig 5). This condition may recapitulate *in vivo* conditions where the small molecule ROCK inhibitor Y-27632 reduces the incidence of contractions [11]. Conversely, increased motor contractility drives filament networks into dense, single aster structures that are similar to ones observed *in vivo* after application of the myosin phosphatase inhibitor Calyculin A [11].

Surprisingly, changes to motor velocity did not drive differences in aster formation. When motors move rapidly (v, 3 μm/s), they are unable to spend sufficient time on filament pairs to pull them into the aster before the motors are trapped (Fig 5). By contrast, the distance motors can search and stretch before detaching (r) strongly affected aster formation (Fig 5). The distance a motor may search as it seeks a binding site on nearby filaments limits the number of filaments that are within range and to a lesser extent, limits the time a motor interacts with filament pairs before detaching (S6 Fig).

In summary, actomyosin aster formation was robust to variations in myosin motor detachment rates, increased attachment rates, increased motor stiffness, and changes in motor speed. However, several factors reduced the ability of asters to form or resulted in multiple small asters; these changes include low motor attachment rate, low motor stiffness, lower motor search or stretch thresholds (r, 0.05 μm or 0.15 μm), high filament turn-over (p_2_, 5 s^-1^), short filaments (L, 0.25 μm or 0.5 μm), and high dynamic viscosity (η, 5 pN s μm^-2^ or 10 pN s μm^-2^). The ability to recruit more filaments into an aster increased with increasing motor stretch (r > 0.6 μm), or increased filament length (L > 1.5 μm). We did not find any conditions that produced oscillating or episodic formation and disassembly of actin asters.

### How might a cell destabilize an existing aster?

**Fig 6:**
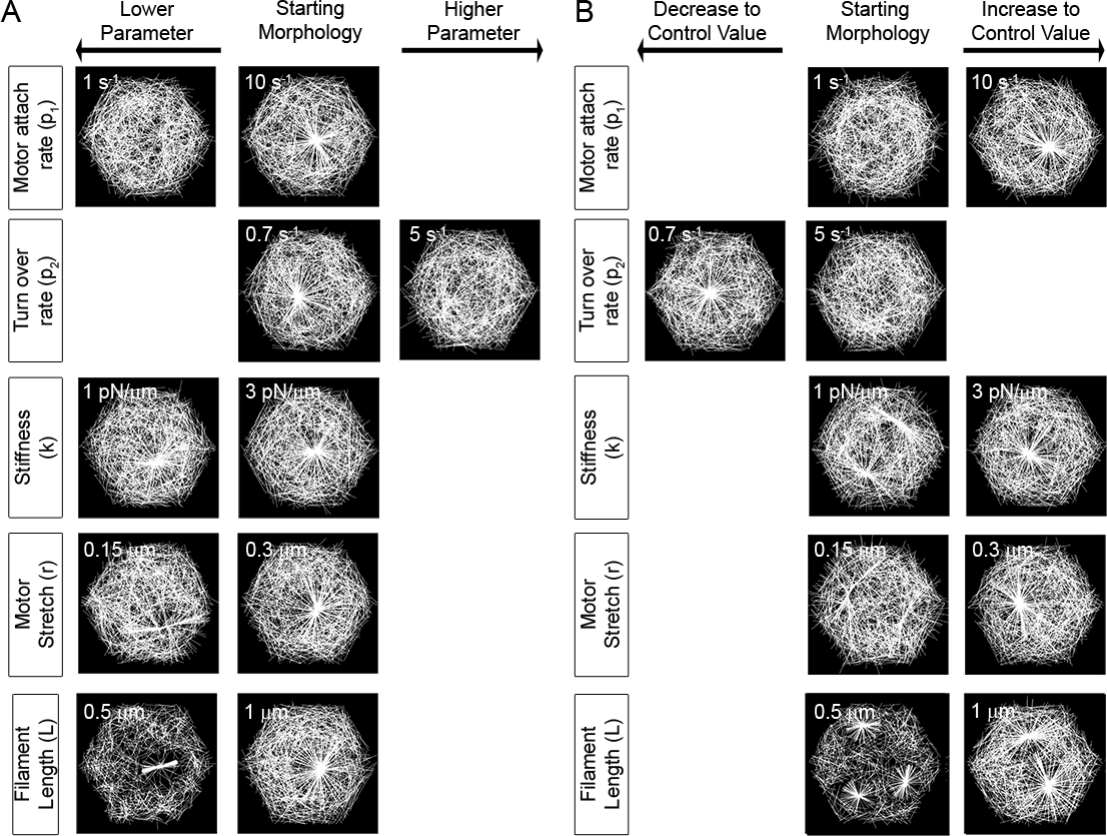
Changing conditions that destabilize or rescue aster formation. (A) To determine whether asters could be destabilized we ran simulations where conditions that produced stable asters were changed to conditions that did not. Conditions for simulations forming quasi-stable asters after 1,000 time steps (center) were changed and the network was followed for 1,000 additional time steps (left or right column). Stable asters are resistant to disruption under most changes except when rates of filament turn-over are increased or when motor attachment rates are reduced. (B) To determine whether asters could be rescued from non-steady states we ran simulations starting with conditions that did not produce stable asters or produced multiple asters before switching to conditions that only formed single asters from initially disorganized networks. Parameters producing the starting morphology after 1,000 time steps are listed in the center columns while the altered parameters are listed in the right (lower) or left (higher) columns. Single aster morphologies could be rescued by changes in motor properties of stiffness or stretch length but could not be rescued by changes in filament length. In all cases the morphology of filament arrays remodeled after 2,000 time steps are shown either under the “Lower Parameter” or “Higher Parameter” columns.

To understand the molecular mechanisms that a cell might use to disassemble an aster, we carried out a series of simulations that began with an aster in steady-state, at which point we changed a parameter and observed the dynamics of the aster over time. In particular we wondered whether asters could transit from one stable state into another stable state. Since there are a few parameter ranges that do not generate single asters by the end of our simulations (t, 1,000 time steps or 10 s; Fig 6), we sought to understand what would occur when conditions were changed so we switched parameters from values that would induce an aster to values that had not previously organized an aster.

To identify conditions that might destabilize an aster we changed conditions that formed stable asters to conditions that had not assembled de novo asters (Figs 4 and 5). As we expected, most cases led to aster disassembly, for instance, decreasing the motor attachment rate (p_1_, from 10 to 1 s^-1^) or increasing the filament turn-over rate (p_2_, from 0.7 to 5 s^-1^) completely annihilated a stable aster. However, some cases of parameter switching resulted in a small remnant of the initial aster (Fig 6A). Such small stable asters were found after decreasing motor stiffness (k, from 3 to 1 pN/μm), reducing the distance a motor can stretch (r, from 0.3 to 0.15 μm), and decreasing filament length (L, from 1 to 0.5 μm). We note that all of these cases resulted in a marked decrease in the number of filaments in the remnant filament aster (S7 Fig). Interestingly, decreasing filament length did not break the stable aster into multiple, smaller asters; at the start of this simulation most motors are already trapped at the aster core and remain sequestered after the parameter switch. Thus, after filaments are shortened, the initial core of the aster persists and continues to trap motors, preventing them from assembling other asters.

We next asked whether we could induce an aster from conditions that had not initially produced a stable aster (Figs 4 and 5). In almost all cases we found parameter switches could generate single stable asters (Fig 6B). An exception was found when increased filament length (L, from 0.5 to 1 μm; S6 Video) generated two asters, however, replicate simulations over longer durations revealed only limited cases where asters merged (S8 Fig, see sims 3, 4, and 9). Our model simulations of aster fusion after changing filament length highlights the rapid changes in structure and dynamics of the cytoskeleton that can be attained by fine tuning actin filament length. Our simulations suggest that cells control episodic aster formation, or punctuated contractions by controlling a limited set of biophysical interactions.

### Simulating *in vitro* dynamics: mechanisms that localize either actin or myosin II motors change actomyosin network morphology

**Fig 7:**
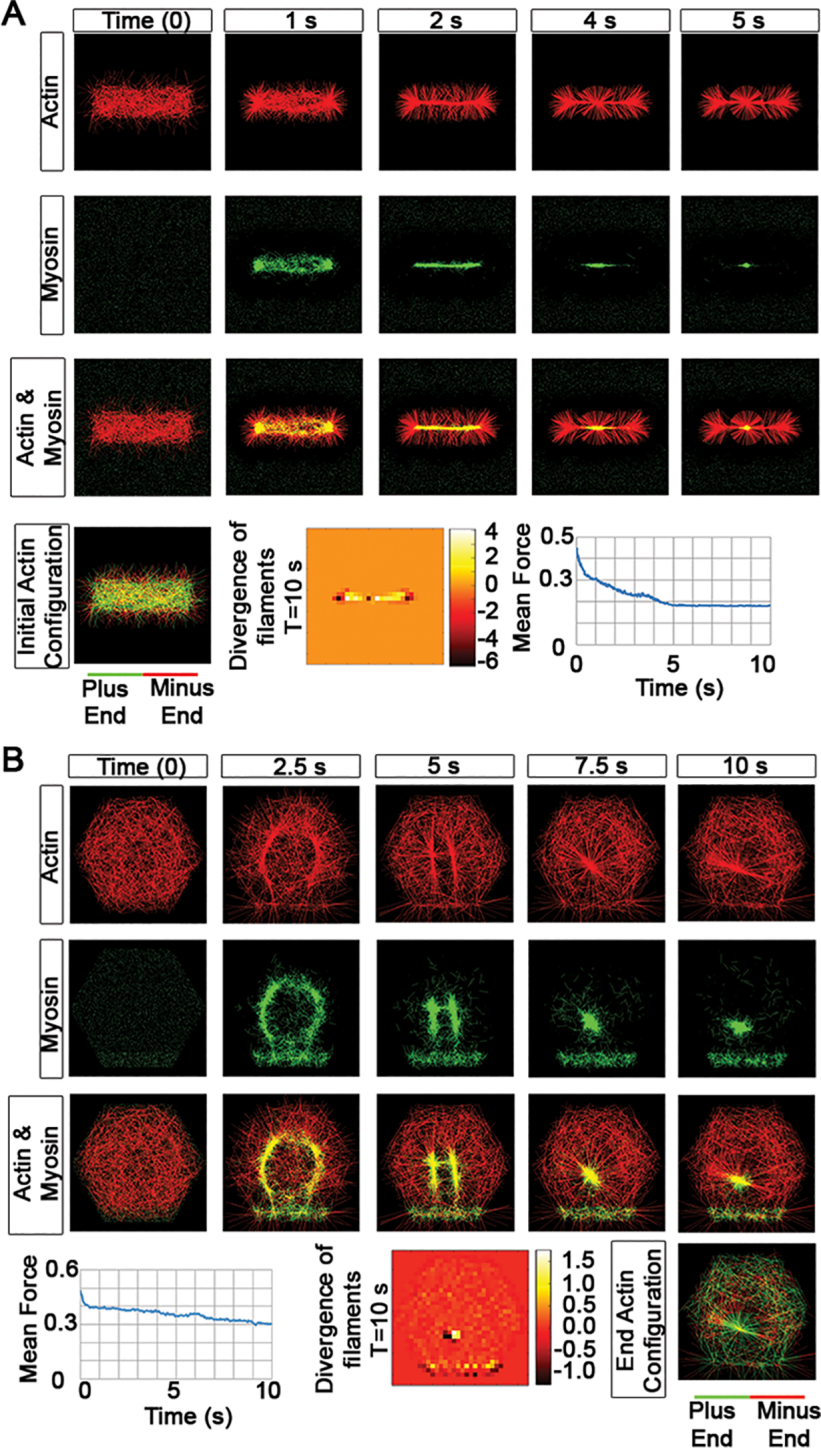
Simulating dynamics of actomyosin networks reconstituted on patterned substrates. (A) Time-evolution of asters formed near a fixed “bar” of actin. The simulation begins with 50% of filaments located entirely inside the bar and 50% of filaments with their minus ends on the bar and plus or barbed ends outside the bar. The density of filaments and motors are the same as previous simulations (1,000 filaments and 5,000 motors). Motors are initially distributed over the entire domain (bar and non-bar). Over time, the motors bind to the filaments and traffic towards filament plus-ends, accumulating within the bar. Abundant motors then contract the F-actin network into an aster. We note: to recreate conditions in the reconstitution studies, filaments in this simulation are stabilized against depolymerization and do not turnover. (B) Time-evolution of asters near tethered motors after a fraction of motors are “tethered” to the bottom eighth of the hexagon. This means that one leg of each motor located in the bottom eighth of the hexagon is fixed in place, leaving the remaining leg free to bind filaments. The freely diffusing motor population interacts with filaments throughout the hexagonal domain. Over time, the fixed-position motors pull asters toward the domain where they are tethered.

In some cases, actomyosin network function may be regulated by scaffolding proteins that localize the actomyosin network to specific sites in the cortex [68–70]. To understand how actomyosin morphology might be shaped by protein localization we simulated cases in which filaments or motors are tethered to specific positions (Fig 7). One such study reconstituted F-actin with Myosin VI and II, and α-actinin and examined the morphology and assembly dynamics of F-actin arrays [26] from patterns of immobilized actomyosin regulators. This study immobilized the actin nucleating protein pWA in a bar pattern on a glass substrate and then added a mixture of G-actin, myosin VI, and ARP2/3. Long actin filaments formed after two minutes with minus-ends confined to the “bar” and plus or barbed ends extending away. To simulate this experiment, we initially localized 50% of the filament minus ends to a rectangular region (Fig 7A, S7 Video) and allowed the remaining filaments and myosin motors to move throughout the entire simulation domain. Motors followed the same rules as our previous simulations; we observed motors reoriented and contracted filaments into an aster distributed over a small subdomain of the bar. Applying our simulation to a more artificial case where filaments were immobilized qualitatively reproduced the actomyosin arrays observed experimentally. Since it is not clear how 3D bulk concentrations in reconstituted systems should be represented by 2D model agents or how fractional ‘activity’ of interacting filaments, motors, and cross-linkers should be represented, we have simulated the experiments using the same domains and number of filaments and motors as our previous examples. Large scale structures up to 1000s of μm across assembled in reconstituted systems involve both early phase of filament nucleation and later phase of filament remodeling. Our simulations are not trying to capture the dynamics of the nucleation phase of the actin filaments, but rather sought to represent the second phase of myosin remodeling actin filaments as might be expected within scaffold-localized subdomains of the cell cortex.

Scaffold proteins can also serve to localize myosin motors and may also regulate actomyosin function in the cortex. In another set of *in vitro* reconstitution studies, myosin VI motors were immobilized to a bar shape on a glass substrate, binding one leg of the motor and leaving the other leg free to bind and move filaments [26]. They observed that the bar of motors pulled a bar of F-actin to the motor location. Our simulation can recreate these same patterns of motor directed filament movements (Fig 7B, S8 Video). To test whether modest levels of immobilized motors could produce the same result, we included free motors in the simulation so that fixed motors accounted for 12.5% of the total (see S2 Text for an explanation of this percentage). We observed the free motor network initially contracts filaments into a characteristic isotropic ring, but that tethered motors subsequently remodel the ring into two asters. The tethered motors then pull the asters toward the lower domain, and finally merge the multiple asters into a single aster over the tethered motor domain (Fig 7B). This result suggests that localized myosin can quickly reposition existing filament networks. We also observed tethered motors can initially deplete filaments in adjacent regions, preventing secondary asters from forming in close proximity to the tethered motors. We also considered the possibility that F-actin binding scaffolds (i.e. anchoring a portion of filaments into a fixed location) may play a role in formation of the actomyosin arrays (see S2 Text; S9 Fig). In this case emergent asters co-align with the position of fixed filaments (S9 Fig). By contrast, a more complex set of aster structures arises from fixing motors. The divergence maps (Fig 7) show that aster-like structures form over both fixed filament and tethered motor sites but a second aster appears in the space adjacent to the sites of tethered motors. Paradoxically, this dynamically stable aster does not move to the site of fixed motors. The plots of the mean motor force show that fixed filament and tethered motor systems (Fig 7) evolve stably along distinct trajectories. In conclusion, by localizing filaments or motors our simulations can qualitatively recreate actomyosin arrays produced in reconstituted systems where filaments or motors are localized by microcontact printing.

### Actin filament cross-linkers change actomyosin network dynamics

Stress fibers, cytokinetic furrows, and adherens junctions all contain parallel arrays of F-actin filaments [71–74]. Yet, the most common structure produced by our simulations is a radial aster. We have not observed stable parallel or anti-parallel arrays or co-aligned filaments; however, we observed many cases where asters form via an intermediate ring-shaped filament array that is composed of co-aligned, anti-parallel filaments. Since the processes that cross-link actin within these transient structures might represent native processes we wondered whether F-actin cross-linkers could stabilize the formation of transiently parallel F-actin arrays.

A wide range of actin bundling or cross-linking proteins have been described [75]. Bundling proteins are a family of actin-binding proteins that bridge and hold together two different actin filaments. These cross-linkers all contain calponin homology (CH) domains that mediate their attachment to F-actin [76]. Bundling proteins vary in their stability, how closely they bind pairs of filaments, the orientation of actin in the bundle, and the specific sites on F-actin that they recognize. Fimbrin and fascin, for example, bind to a pair of filaments with the same polar direction, at binding sites every 3.5 to 5 actin subunits, and keep the F-actin pairs 10 nm apart [75]. In addition, fimbrin and fascin limit myosin motors access to bundled filaments. By contrast, α-actinin, another cross-linker binds and orients filaments and maintains a spacing of 40 nm, which is thought to allow myosin motors to interact and contract the network [74, 77]. Filamin, another F-actin cross-linker, forms a v-shaped link and binds at the cross-over point between orthogonal filaments. Networks cross-linked by filamin are relatively flexible and can deform [74].

**Fig 8:**
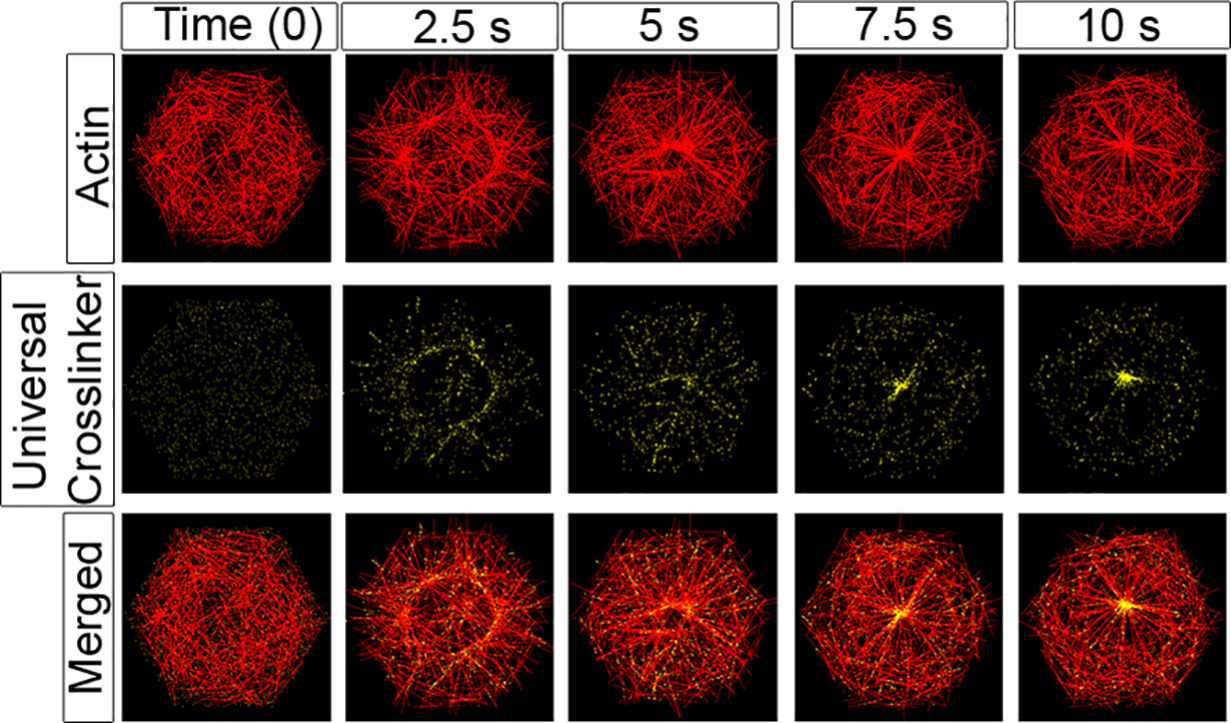
Generic actin cross-linkers slow but do not prevent aster formation. Time-evolution of filaments (red), actin cross-linkers (yellow; 1,250) and motors (3,750, not shown) show cross linkers do not inhibit formation of asters. Cross-linkers bind at the same rate as motors (p_0_) to filament pairs that are oriented within 22.5° (π/8) of each other and no more than 40 nm apart. The cross linkers bind to the shortest distance between candidate filament pairs, and remain fixed. The merged images in the bottom row show the formation of an aster over the time course. Biophysical parameters guiding filaments and motors in these simulations are the standard set (see S1 Table for details).

To model fimbrin, fascin, α-actinin and the more general class of CH containing F-actin cross-linking proteins we introduce to our simulations a population of cross-linkers that only bind filament pairs that are co-oriented within a range of angles up to π/8 (22.5°). We simulate cross-linkers by letting the cross-linker search and bind filament pairs in the way we simulate motor binding. Once bound we allowed cross-linkers to stretch to 40 nm, similar to the distance spanned by myosin filaments [78]. As the case with motors, multiple cross-linkers can attach to each filament and once bound to a pair of filaments the cross-linker exerts spring-like forces at the binding site on each filament. While different cross-linkers may differ in their ability to interfere with motor processivity, for simplicity, we assume that motors can pass through any bound cross-linker. Surprisingly, simulations with cross-linkers do not produce significant numbers of parallel filaments, but do slow aster formation (Fig 8, S9 Video). We next asked whether cross-linkers could alter aster formation and found that only a three-fold excess of cross-linkers to motors (3,750, compared 1250 motors) could inhibit asters from forming (S10 Fig). Since simulations with cross-linkers generate transient ring alignment of filaments rather than parallel stress-fiber-like arrays, we hypothesize that bundling cross-linkers might stabilize parallel arrays through other mechanisms, such as altering filament turn over or inhibiting motor-mediated remodeling.

**Fig 9:**
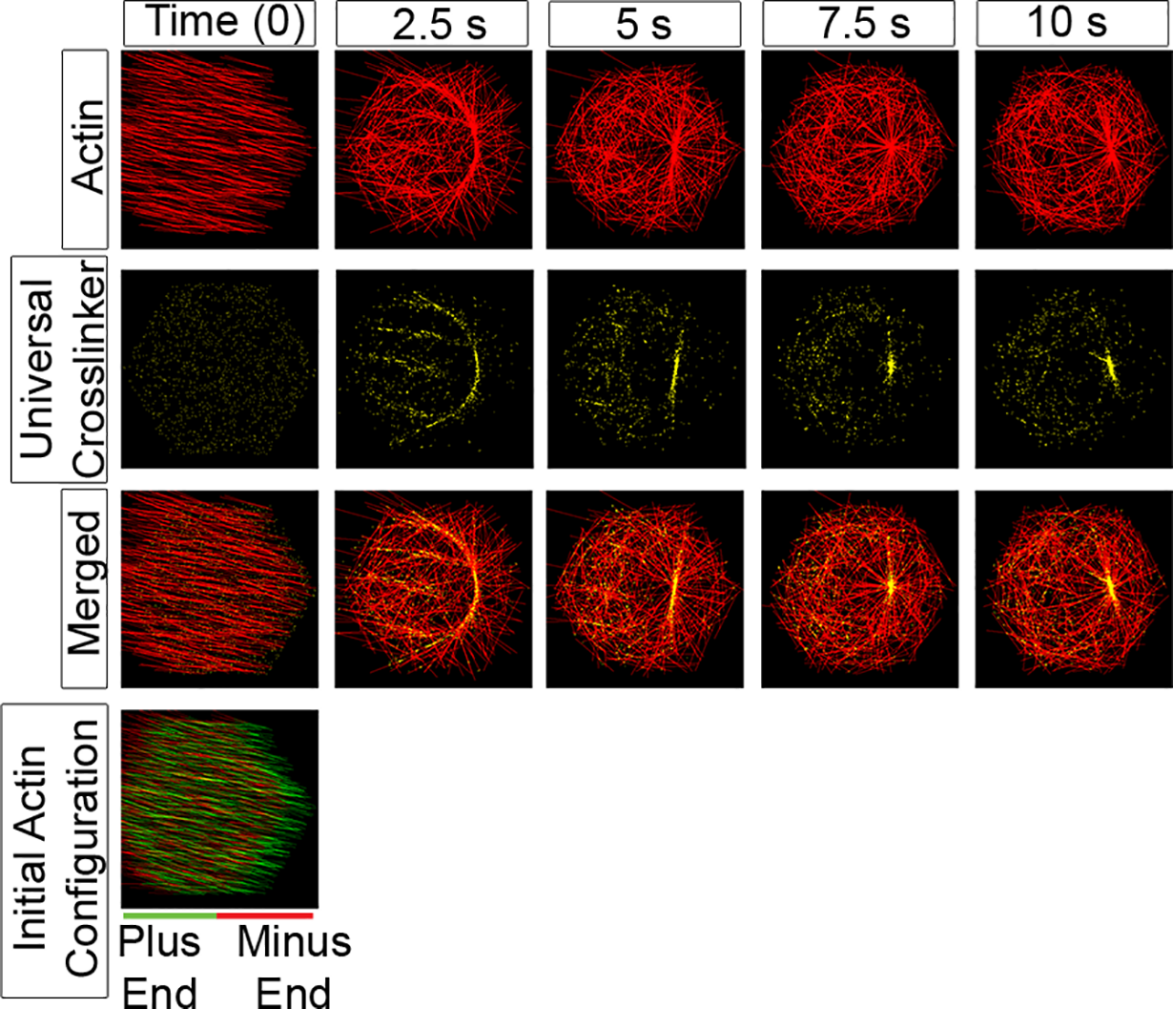
Generic cross linkers do not stabilize aligned F-actin arrays. To test whether cross-linkers (yellow) can stabilize previously aligned filaments (red) and inhibit aster formation we prepared an aligned array of F-actin for the start of a simulation. Filament plus-ends are initially distributed randomly throughout the hexagon with their orientation between 0 and 22.5°. Cross-linkers bind at the same rate as motors (p_0_) to filament pairs that are within π/8 degrees of each other and no more than 40 nm apart. The simulation was run with 1,250 cross-linkers and 3,750 myosin motors (not shown). The filament asters form, albeit with distinct intermediate morphologies despite the prior alignment of filaments. Biophysical parameters guiding filaments and motors in these simulations are the standard set (see S1 Table for details).

Since parallel filaments do not emerge from the addition of cross-linkers, we asked whether actin cross-linking proteins might stabilize initially parallel filaments. To test this we included cross-linkers within a simulation that started with aligned filaments (within 22°; Fig 9). By pre-forming parallel filament arrays we expected cross-linkers (1,250) would bind filaments and allow filament arrays to resist reorientation by motors (3,750). Instead, we found motors were still able to reorient and remodel filament arrays into asters (Fig 9). In conclusion, the addition of actin cross-linking proteins to our model can slow or prevent the central aster from forming. Tight parallel bundles of filaments as seen in stress fibers, for example, do not emerge for our model even in cases where excess parallel binding cross-linkers are added.

## Discussion

**Fig 10:**
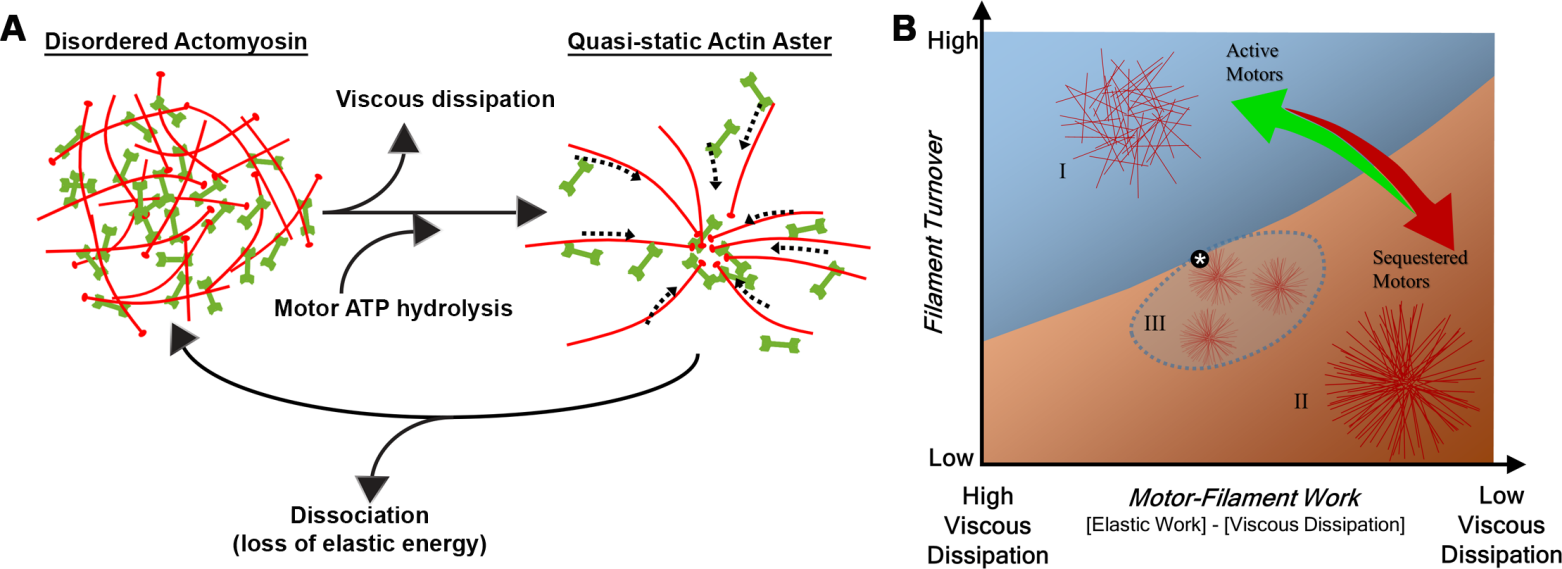
Qualitative maps of phase transitions in actomyosin arrays. (a) Proposed model for how disordered actomyosin arrays transition to quasi-static actin asters. Viscous dissipation leaves the system as motor ATP hydrolysis generates reorganization of the actin network. For a quasi-static actin aster to revert back to a disordered state, we believe there is a dissociation of the network resulting from a loss of elastic energy. (b) Phase transitions in parametric space are determined by physical principles of work energy of a dissipative system of filaments, motors, and their viscous environment. For instance, high rates of force production lead to greater losses to viscous dissipation and less contribution to elastic remodeling of the filament array. Aster morphologies (region II) can emerge from disordered arrays (region I) via several mechanisms including reduced filament turnover, reduction of viscous losses, or increased motor-work. In special cases where filaments are shortened (dashed domain, region III) multiple asters can form. Multiple asters are also sensitive to work energy and can merge into single asters (transition to region II) or disperse into disordered arrays (transition to region I) under similar conditions that mediate aster/no aster transitions. The asterisk indicates the location of our ‘standard’ parameter set leading to a single robust aster.

Our mesoscale molecular dynamics model recapitulates native cortical actomyosin dynamics by simulating myosin II motor search, binding to actin filaments, and polarized motor movements that contract and remodel filament arrays into aster-like structures. Asters emerge as filaments undergo "polarity-sorting" [51] and motors are transported to and trapped within aster cores. Our findings confirm previous 2D studies that have simulated aster emergence from simple motor-filament interactions [51, 55]. We have further confirmed that increasing rates of actin turnover can inhibit aster formation [55] in accordance with predictions from our earlier rotational model [47] and a simplified 1D model (S3 Text and S11 Fig). As the rate of filament turnover increased in the 1D model, the number and alignment of filaments in the contraction and the force generated by the motors increased. Although the geometry and boundary conditions of the 1D models differ significantly from our current model, in 2D we observe asters become less dense as filament turn-over rates increase (Fig 4; Fig 10) and that steady state force exerted by the motors also increases (S12 Fig). Complex patterns of motor transport and activity emerge from our model without an explicit feedback mechanism such as a catch-bond. In the absence of filament turnover, motors quickly align filaments into arrays which in turn sequester motors, removing the motors from active force production. As filament turnover increases the compactness of the aster is reduced and fewer motors are sequestered leading to higher levels of force production (S13 Fig; S12 Video).

Disordered, single aster, and multiple aster states of F-actin in the cortex and the transitions between them can be understood in terms of the work-energy principle that shapes the network and the filament turnover processes that destabilize the network (Fig 10). Filaments and motors form a dissipative system that remodels as a consequence of both elastic work and losses due to viscous dissipation (Fig 10A). ATP hydrolysis by filament bound motors is the ultimate power source responsible for driving filaments to remodel but the dynamic time-scale and morphology of the array that emerges depends on reactive dissipative forces that resist movement of the filaments and transport of motors along those filaments. In our simulations we can track motors states as they bind filaments and segregate from filaments; these evolving changes in motor association with filaments first increases force production that is then dissipated as filaments sort into asters (S14 Fig). While work stored in an array's elastic energy can be dissipated as motor coupled filaments are depolymerized, our calculations suggest this form of dissipation is negligible compared to the energy lost to viscous dissipation (Fig 10B; S14 Fig). Whereas previous studies have focused on force production and transmission through the elastic network we propose new biophysical studies on frictional forces operating in the cortex. Interestingly, moderate rates of actin turnover enhance the rate of frictional forces by maintaining high rates of filament motion. The process of aster formation is commonly associated with cell shape change and force production but our model suggests that by trapping motors in their core asters deplete motors from other regions of the cortex and are indicators of low levels of contractility.

The processes that drive both transient F-actin rings and dense plus-end rich aster cores in our simulations could be tested by assays of actin polarity and myosin II motor distribution. A recent simulation using an approach similar to ours has suggested either cross-linkers or branched F-actin networks are required to stabilize actin rings [54]. However, in our simulations ring structures emerge transiently as asters form and do not require that we initiate the simulation with a ring-structure, cross-linkers, or branching factors. A previous, simpler version of our model [79] also produced ring-like arrays of F-actin when boundary conditions were imposed that mimicked stiff surroundings of cell-cell or cell-ECM junctions. One possible reason our results differ from Ennomani et al. [54] is the parameters used to control motor activity. Motors in Ennomani et al. are stiffer than ours (100 pN/μm versus 3 pN/μm; Table 2), with a rupture force of 3.65 pN compared to our maximum motor exerted force of 0.9 pN. Additionally, our simulations involve more motors (5,000 compared to Ennomani et al.’s 2,000 motors) that bind F-actin more frequently (10 s-1 versus Ennomani et al.’s 5 s-1) and move more quickly (1 μm/s versus Ennomani et al.’s 0.3 μm/s). Thus, each myosin motor implemented in our model does more work in remodeling filament arrays than those in Ennomani et al., even though the maximum force exerted by our motors is four-fold lower. Fewer active motors and highly stable filament cross-linkers may be responsible for stabilizing filaments into persistent ring-like arrays.

Episodic actomyosin contractions of the cell cortex suggest the need for episodic mechanical or biochemical signaling to drive cycles of aster assembly and disassembly. *In vivo* cortical actomyosin networks can exhibit pulsatile contractions whereas our 2D models result in a steady state contraction. Rather than capturing the dynamic signaling environment of the cell, our 2D simulations recreate reconstituted actomyosin gels which contract in a similar manner to produce a large stable structure [25, 31, 80, 81]. Asters generated in our simulations are highly stable and require specific changes in the biophysical conditions for disassembly (Fig 10). By switching model parameters after a steady state had been achieved, we identified several strategies that might disrupt or trigger formation of an aster including: 1) changing the motor attachment rate tenfold, 2) changing the motor stretch by half, and 3) changing the filament length by half. Our model predictions suggest quantitative studies of aster dynamics after mutant myosin II motors, or factors, such as capping factor to regulate the length of F-actin in the cortex, or signaling pathways that modulate these effectors are targeted to the cell cortex.

Alternatively, it has been suggested that cycles of contraction and relaxation in the actin cortex might reflect F-actin disassembly as highly compressed F-actin filaments bend and are severed [82]. A recent 2D microstructural model of contracting filament networks demonstrated that tensed networks can drive filament bending that could be sufficient for severing but do so well before recognizable asters form [48]. We did not incorporate mechanisms to sever or depolymerize filaments under tension, however, we note that F-actin within stable asters experience very low forces since most motors are segregated from potentially productive pairs of filaments. Instead, F-actin arrays in our simulations generate highest tension early in aster formation as broadly distributed motors remodel filament arrays. Interestingly, both models confirm findings from our earlier 1D model that ordered arrays generate the lowest levels of force [47]. Thus, feedback through tension-mediated filament disassembly appears unlikely to generate cycles of cortical aster assembly and disassembly.

To investigate mechanisms that might stabilize or orient filament arrays we introduced actin cross-linking proteins into our simulations (Fig 8). In the light of prior modeling studies [51, 53, 55, 83] we were surprised that cross-linkers did not stabilize oriented filaments in our simulations. Instead, we found that filaments cross-linked at a preferred angle of orientation are not sufficiently stable against motor-mediated remodeling or under high rates of filament turnover. Steric locking of filament arrays, allowed in Mak et al. [55], but not present in our simulations may serve to increase stress and lock motors into rigor states and to stabilize the F-actin array. *In vivo* cross-linkers may inhibit motor access or drive motors to dissociate from filaments and substantially reduce the long range transport of motors. Our simulations do not account for the ability of some cross-linkers to block motor access [84], instead our simulated motors can walk past the cross linkers as they do in α-actinin cross-linked filaments. Motor-blocking cross-linkers could additionally lower the dwell time of the motor on a filament and slow or reduce polarity sorting of filaments. To test the role of cross-linking proteins more realistically would require inhibiting motor processivity, varying binding kinetics, cross-linker length, and the ability of cross-linkers to hold filaments at fixed angles to match the biophysical and biochemical behaviors of specific F-actin cross-linking proteins.

There is growing adoption of microscopic computational models for studying the complex interactions that shape the cytoskeleton [32, 85]. Our model of cortical actomyosin dynamics shares many features with recent mesoscale molecular simulations of actomyosin arrays but extends those models with constraints that are unique to the thin cortex of embryonic cells. Mesoscale molecular models are advantageous because they can examine the influence of external mechanical conditions on disordered filament network arrays. Our model is formally similar to other simulations based on Langevin dynamics (e.g. Cytosim [53, 54]), and like these other models, incorporates experimentally determined parameters that describe biophysical behaviors of actomyosin networks. Differences in model construction, initial conditions, boundary conditions, and implementation of biochemical and biophysical processes make it challenging to directly compare predictions of these models, however, these computational models reveal how actin filament polarity, actin motors, and actin turnover and polymerization drive the emergence of distinctive actin array morphologies. The results of our 2D model qualitatively parallel findings from a simpler 1D rotational model [47] and a 1D linear model (S3 Text and S11 Fig) where higher rates of F-actin turn-over affected, but did not totally inhibit formation of a quasi-steady state contractile filament array. Although the geometry and boundary conditions of the 1D models differ significantly from our current model, in 2D we observe asters that become less dense as filament turn-over rates are increased (Fig 4; Fig 10) with increasing levels of steady state force and network work (S14 Fig).

Increasing computational requirements for microstructural simulations led us to seek simplifications that would allow us to capture the key elements that shape actomyosin arrays within the cell cortex and aid in understanding how cells might disassemble actin arrays or spatially control morphologies through localization of actin-binding proteins. Our simplified model of actomyosin interactions have provided novel insights into the relative importance of elastic energy storage and viscous dissipation that suggests filament frictional forces may play a key role in transmitting work from actomyosin networks to cell shape and tissue mechanics. Complex patterns of motor transport and activity emerge from our model without imposing an explicit feedback mechanism. In the absence of filament turnover motors quickly align filaments into arrays which in turn sequester motors, removing them from active force production. As filament turnover increases the compactness of the aster is reduced and fewer motors are sequestered leading to higher levels of force production. In addition, aster-formation becomes less likely as filament turnover increases since motors are not able to coordinate large scale remodeling of the filament array. The asters produced by our model resemble those generated by other groups, however we are able to produce asters without explicit feedback mechanisms [86].

Our model implements *search*, *capture*, *remodel*, *and traffic* processes by analogy with the 'search, capture, pull, and release' model developed to describe the involvement of actomyosin in the cytokinetic furrow of fission yeast [46]. In our model, polarized arrays of filaments transport motors towards the filament plus-ends, and directional alignment of filament arrays serves to transport motors, sequestering them within the cortex. Together, these processes generate a stable aster with filament plus-ends concentrated in a small central region, which depletes motors from regions with high minus-end densities and traps motors in the aster center. Motor trafficking on oriented filament arrays results in motors becoming trapped in filament plus-end “cages” because they are unable to move out of the aster center; motors that escape the cage via diffusion quickly bind new filaments that direct them back to the center of the aster. A key prediction of our model is that filaments within contracted actin asters are polarized with their plus-ends embedded in the aster center.

The dynamics of actomyosin arrays simulated here may be analogous to a case of microtubule-kinesin arrays which remodel into “pineapple” morphologies and multiple aster-like arrays with centrally located plus-ends [87]. Similar to microtubule-kinesin arrays, our model predicts that F-actin plus-end localization and myosin trafficking play critical roles in the formation of asters. The polarity of F-actin or locations of myosin II motors within these dynamic contractions have not yet been resolved but actin filament polarity might be revealed in fixed cells since there are no methods to visualize F-actin plus-ends in live cells. Identifying the *in vivo* polarity of F-actin arrays predicted by our model will require improved experimental techniques. F-actin plus-end trackers, analogous to CLIP-170-GFP used to track microtubule plus-ends [88], are not currently available for studying F-actin plus-end dynamics.

Our model can be extended to include more biophysical and biochemical realism. For instance, simulated filaments have a fixed length and a single parameter controlling turn-over. This has allowed us to test roles of filament density and length independent of turn-over rates. However, in the cell, filament turnover, length, and density would be closely coupled; higher depolymerization rates may generate shorter actin filaments at lower density. Additionally, filaments in our model do not interact physically with one another. Filaments can pass through each other as they rearrange or can be packed at high density without nematic or lateral association effects on their orientation. As a model utilizing Langevin dynamics, our model can be easily extended to include complex programs of polymerization (e.g. [89]), steric interactions (e.g. [53]), or other biochemical or mechanical interactions. Furthermore, our model makes predictions about the roles of myosin stiffness and filament orientation that would be difficult to examine without fine-grained experimental control. As actomyosin models continue to evolve they will be more able to guide and interpret *in vivo* studies.

## Materials and methods

### Ethics statement

*Xenopus laevis* aquatic frogs used in this study were cared for according to principles and standard operating procedures established by the University of Pittsburgh IACUC protocol #15025409 (PHS Assurance Number: A3187-01).

### 2D model development

Planar arrays of actomyosin within the cell cortex are confined to a 0.2 μm thick volume beneath the plasma membrane [90] which we simulate with a 2D array of discrete agents representing F-actin filaments (referred to as filaments) and non-muscle myosin mini-thick filaments (referred to as motors). To capture the dynamics of these networks we use experimentally determined biophysical parameters or ranges of parameters (S1 Table). Motors connecting two filaments apply forces to those filaments. Forces are then summed and drive filaments to rearrange within a viscous media. Filaments, motors and cross-linker dynamics and movements are carried out within a Monte Carlo framework. In the sections below we describe the model rationale and implementation details.

### F-actin

In cells, filamentous actin (microfilaments or F-actin) can vary in length from a few G-actin subunits to more than 10 μm. F-actin exhibits a distinct polarity of plus or barbed and minus- ends with distinctive polymerization rates. F-actin can polymerize or depolymerize depending on the concentrations of G-actin and polymerization factors [91]. Filament polarity also directs myosin motor movement to the filament plus-end. These fundamental properties of F-actin are likely shared throughout most living cells [92].

Filaments in our simulations are represented as polarized cylindrical rods with a fixed length of 1 μm. Because the scale of our simulations are small compared to large *in vitro* actin filament arrays, we have modeled F-actin as rigid and not semi-flexible. Initially our simulation places filaments at random positions (*x*_*i*_, *y*_*i*_) and angles (*θ*_*i*_) within a hexagonal boundary. The filaments then move in response to forces exerted by attached, stretched motor complexes. In the cell, F-actin length can grow and shrink through polymerization and depolymerization (Fig 2A). A fully realized model of F-actin would require tracking filament subunits (e.g. G-actin) and their differential addition and removal from the plus and minus-ends of F-actin. However, for simplicity we represent filament polymerization processes in the simulation with a single turn-over rate that removes a randomly chosen filament and adds a new filament in a random location and orientation in one time step. Any motors attached to a filament that turns over detach. Such stochastic events of filament depolymerization (for rates see S1 Table) introduce an element of spatial "noise" similar to that implemented in our earlier model [47].

One aspect of filament dynamics is treadmilling where the addition of new G-actin monomers to the plus or barbed end of the existing F-actin is faster than the subtraction of monomer from the minus end causing F-actin to “move” in the direction of the plus or barbed end. In order to determine how fast F-actin would move due to treadmilling, we considered the rate of *in vivo* treadmilling from Selve and Wegner [93] of 0.21 molecules/second, and the typical size of a G-actin monomer of 4–7 nm. The forward velocity of any F-actin due to treadmilling would then be 8.4 x 10^−4^ to 1.47 x 10^−3^ μm/s. Our simulation time step is 0.01 s, which would mean a filament would move between 8.4 x 10^−6^ to 1.47 x 10^−5^ μm, which is small so we have assumed no contribution of treadmilling to filament movement in the simulation.

### Myosin filaments

Multiple non-muscle myosin heavy chain and regulatory light chains are associated with F-actin as myosin filaments (also known as mini thick filaments, [94]). Myosin filaments take a variety of forms but are generally composed of 15 to 30 myosin motor heads at either end of a bipolar myosin filament (Lecuit et al, 2011). The stiffness of myosin filaments have been measured and they can generate a range of forces from 240 to 21,000 pN [95–97]. In our model, each end of the motor can independently bind to an actin filament and processively walk to the filament plus-end (Fig 2B). If the two ends of a motor move apart on two filaments and separate, they exert a spring-like force on the two filaments at their respective points of attachment (Fig 2C and 2D). For simplicity, simulated motors, representing single mini thick-filaments, have 0 rest length. As they stretch between two filaments they exert equal and oppositely directed spring forces at their attachments on each filament. The maximum force a motor complex can exert is based on the motor’s spring stiffness and its maximal stretch (S1 Table).

Motor movement along each filament is implicitly due to ATP-dependent cross bridge cycling of myosin motors within the myosin filament. We do not explicitly represent the biophysics of this cross bridge cycling because the time steps in the simulation are longer than the time scale of cross bridge cycling. Instead, we simulate motor processivity with a constant, plus-end directed motor velocity.

We have modeled filament associated motors as non-muscle myosin II mini-thick filaments. A single motor operates as a two headed Hookean spring. The heads on either end of mini-thick filament interact with actin filaments and extend as a spring with a spring stiffness constant of 3 pN/μm. Previous work [98] had performed *in vitro* assessments of the spring stiffness for individual non-muscle myosin II mini-thick filaments as being around 300 pN/μm, but with force measurements of 1–10 pN. Motors can exist in three states, either free diffusing, attached to a single filament, or attached to two filaments. Motors only exert forces when they are bound to two filaments. Attachment and detachment of a motor to a filament is probabilistic with independent rates. Free diffusing motors may bind to a pair of filaments located within their search radius, and they will attach to two different filaments if more than one filament is within the search radius. This radius is based on the size of a free myosin mini thick filament (~300 nm). This search length also serves as the maximum stretch a motor is allowed (Fig 2E). If only one filament is near the motor, the motor attaches to that one filament and processively moves toward the filament plus-end. At each time step a motor bound to one filament will seek a second filament within the defined search radius. If a filament is found, the motor binds stochastically according to the attachment rate. Motors bound to one or more filaments detach from a filament once the motor reaches the plus-end of the filament, is stretched past its threshold stretch radius, or is stochastically selected to detach according to the detachment rate. Motors detached from single filaments join either the free diffusing pool or, if remaining attached to another filament, move toward the plus-end of the bound filament.

### Actomyosin interactions

In order to recapitulate actomyosin dynamics within the cell cortex we extended our previous rotational model of filament motor interactions [47] to a two-dimensional domain where additional biophysical modeling allowed free filament movement (see Table 1). Movements of motors and filaments are advanced according to finite difference scheme. With each time step, we first calculated the forces from all motors acting on filament *i*. I.e. if a motor *j* is attached to a filament *i*, then it has a nonzero length (*len*), otherwise, motor *j* has a zero length.

$$\begin{array}{l} a_{j}=x_{i}+len_{j}*\cos\left(\theta \right)\\ b_{j}=y_{i}+len_{j}*\sin\left(\theta \right) \end{array}$$ (1)

**Table 1:** Symbols used in model equations.

Symbol	Description
*N*	Number of filaments
*M*	Number of motors
(*x*_*i*_,*y*_*i*_)	Position of center of mass of filament *i*
*θ*_*i*_	Angle of orientation of filament *i*
(*a*_*j*_,*b*_*j*_)	Coordinates where motor *j* is attached to filament *i*
(*A*_*j*_,*B*_*j*_)	Coordinates of the end of stretched motor *j*
*len*_*j*_	The distance from the center of mass of filament *i* to the attachment point of motor *j*
*k*	Spring stiffness constant for motors
*L*	Length of the filament
*η*	Dynamic viscosity of the media
*p*	Ratio of length of filament to its diameter
*γ*_*perp*_ = 0.84 *γ*_*par*_ = 0.114 *γ*_*rot*_ = −0.662	Viscous drag shape coefficients when *p* = ∞

The resulting forces for each motor on each filament are then calculated:

$$F_{j}=\left[\begin{array}{c} k*\left(A_{j}-a_{j}\right)\\ k*\left(B_{j}-b_{j}\right) \end{array}\right]$$ (2)

We transform to the parallel and perpendicular coordinate system of the filament using the following rotation matrix:

$$\begin{array}{l} XR_{i}=\left[\begin{array}{cc} \cos\left(\theta_{i}\right) & \sin\left(\theta_{i}\right)\\ -\sin\left(\theta_{i}\right) & \cos\left(\theta_{i}\right) \end{array}\right]\left[\begin{array}{c} x_{i}\\ y_{i} \end{array}\right]\\ FR_{j}=\left[\begin{array}{cc} \cos\left(\theta_{i}\right) & \sin\left(\theta_{i}\right)\\ -\sin\left(\theta_{i}\right) & \cos\left(\theta_{i}\right) \end{array}\right]F_{j} \end{array}$$ (3)

We then update the positions of the filament by updating the center of mass based on the parallel and perpendicular translations, and the angle of orientation by the applied torque. First, we determine frictional drag coefficients for the cylinder in each of these directions. To calculate viscous drag, we use the following drag constants, where *p* is the ratio of the length (*L*) to diameter (*di*) of the cylinder, and *γ*_*perp*_ = 0.84, *γ*_*par*_ = 0.114 and *γ*_*rot*_ = −0.662 are constants when *p* = ∞ [99]. We assume the filaments experience high shear, so the dynamic viscosity, *η*, is higher than water.

$$\begin{array}{c} \Gamma_{perp}=\frac{4\pi \eta L}{\log p+\gamma_{perp}}\\ \Gamma_{par}=\frac{2\pi \eta L}{\log p+\gamma_{par}}\\ \Gamma_{rot}=\frac{\frac{1}{3}\pi \eta L^{3}}{\log p+\gamma_{rot}} \end{array}$$ (4)

Next, we update the positions of the filaments by updating the center of mass based on the parallel and perpendicular translations, and the angle of orientation by the applied torque.

$$\begin{array}{c} XR_{n_{i}}\left(1\right)=XR_{i}\left(1\right)+dt*\frac{1}{\Gamma_{par}}\sum_{j}FR_{j}\left(1\right)\\ XR_{n_{i}}(2)=XR_{i}(2)+dt*\frac{1}{\Gamma_{perp}}\sum_{j}FR_{j}(2)\\ \theta_{n_{i}}=\theta_{i}+dt*\frac{1}{\Gamma_{rot}}\sum_{j}len_{j}*FR_{j}(2) \end{array}$$ (5)

We then transform the updated positions for the filament back into the original coordinate system using the inverse rotation matrix.

$$\begin{array}{c} \left[\begin{array}{c} x_{i}\\ y_{i} \end{array}\right]=\left[\begin{array}{cc} \cos\left(\theta_{i}\right) & -\sin\left(\theta_{i}\right)\\ \sin\left(\theta_{i}\right) & \cos\left(\theta_{i}\right) \end{array}\right]XR_{n}{}_{i}\\ \theta_{i}=\theta_{n_{i}} \end{array}$$ (6)

### Boundary conditions

Simulated actomyosin interactions occur within an open 2D hexagonal domain. The thickness of cortical F-actin in *Xenopus* embryonic cells [11] and cultured cells [90] is approximately 0.2 μm. Cortical actomyosin arrays are essentially planar when compared to the 20 to 40 μm diameter the *Xenopus* embryonic cells. Unlike approaches utilizing periodic boundary condition, filaments or motors that move out of the hexagonal domain are randomly re-inserted into the domain. Parallel filaments are not attracted to one another and do not interact sterically but instead slide through and past each other.

### Simulation statistics and image analysis techniques

Simulations were implemented in Matlab (Mathworks, Inc, Natick, MA) and the plots carried out in ImageJ [100]. For most cases, the simulations were run for 1,000 time steps at a step size of 0.01s, which translates to approximately 10 seconds (*in vivo* contractions in *Xenopus* typically form in 45 seconds [11]). This time was chosen for comparison because aster stability in the standard case had been reached. We confirmed aster stability through inspection of long duration simulations (S5 Fig, S15 Fig, and S3 Table). Aster formation is also accompanied by a reduction in mean motor force (S2 Fig). Mean force decreases as more motors become trapped in the center of the aster and spend less time remodeling or aligning filaments.

Model time-lapse sequences simulate fluorescence microscope images by additively increasing intensity of a pixel when more than one filament or motor are present. Single image frames, kymographs, and intensity profiles were generated from simulated time-lapse sequences using ImageJ. Mean intensity profile plots were generated by determining a region of interest, in the case of Fig 3B the ROI was a circle, and then calculating the mean intensity within the ROI over time.

To quantify aster structural evolution, we have employed two strategies. First, using image analysis tools previously used to assess F-actin networks in vivo [11, 17, 63], and using divergence of filament orientations to identify how filaments are oriented during aster evolution (S1 Text). The first method segments actin-dense regions and can track the number, duration, and movements of actin-asters. Quantitation with this method shows that large stable asters form and persist in a quasi-stable configuration, and identifies small transient asters that form at the periphery before being drawn into the large central aster (Fig 3G; S3 Fig).

Our second strategy to assess filament networks uses exact locations of filaments and motors to calculate the mean motor generated forces and clustering of filament plus-ends over time. Asters were identified by the divergence of filaments in a small grid divided into L/8 size boxes. Our algorithm determined which filament plus-ends were located within the box, and vector orientations for each filament within the box were added to determine a summed orientation for each box ($\vec{V_{i,j}}=[V_{x_{i,j}},V_{y_{i,j}}]$). We then used a second order derivative approximation to determine the divergence in x (*divX*_*i*,*j*_) and y (*divY*_*i*,*j*_) for each box. The total divergence is the sum of divergence in x and y and was plotted using a pseudocolor scale to highlight where a sharp transition from low divergence to high divergence is spatially located for any time during the simulation. If filaments are randomly distributed the divergence will be 0, but for an aster formation, the divergence will be negative at the center of the aster and then sharply transition to positive in the area surrounding the center of the aster.

$$\begin{array}{c} divX_{i,j}=\frac{V_{x_{i,j+1}}-V_{x_{i,j-1}}}{2(\frac{L}{8})}\\ divY_{i,j}=\frac{V_{y_{i,j+1}}-V_{y_{i,j-1}}}{2(\frac{L}{8})}\\ DIV_{i-1,j-1}=divX_{i,j}+divY_{i,j} \end{array}$$

### Actomyosin model formulation

Microstructural simulations of cytoskeletal dynamics provide unique insights into the fundamental biophysical and biochemical processes that guide cell shape and morphogenesis [85]. Several papers have been published that use similar modeling approaches to those we present in this paper [50, 51, 54, 55]. Two of these papers [54, 55] apply Langevin dynamics to microstructural models of F-actin and myosin motors similar to our own. Both models find F-actin contractions, either through motor-based contraction [55] or through defined filament cross-linkers [54]. The mesoscopic structures these two models produce, e.g. actin asters [55] and ring-like structures [54], are similar to the actin asters and transient ring structures produced in our simulations.

The first of these models by Mak, et al. [55] simulates actomyosin networks with actin filaments, myosin motors, and actin cross-linking proteins. By comparison to our minimal representation, the Mak model includes a diverse array of complex interactions including specialized catch-bonds in myosin motors, steric interactions between filaments, and filament treadmilling. In addition, actin binding proteins that cross-link filaments are included and allowed to stabilize the filament array prior to initiation of motor activity. Despite these differences in formulation, both our approach and that by Mak et al. reveal emergence of actomyosin asters that are inhibited by increasing rates of filament turnover. However, implementation differences in motor activity can produce different results as cross-linking proteins are incorporated. In our models, cross-linking proteins slow the emergence of asters but do not completely block their formation unless provided in vast excess. We suspect this difference reflects differences in motor function and differences in the initial conditions. Mak, et al. have chosen to include motile motors that exert forces on actin filaments through an extremely stiff connection between the myosin II tail (or backbone) domains as they bind two filaments. Like our model, myosin becomes extended between the pair of bound filaments and force increases. In our model, the motors can dissociate stochastically, dissociate once they reach to plus-end, or once they extend to their threshold. By contrast motors in Mak slow and lock in a rigor state once they reach a threshold stall force (5.7 pN; Table 2). The maximum stall force is not much different than maximum exerted force of 0.9 pN but the subsequent stalling of motor processivity may lead to more stable filament networks and increase the apparent cross-linker density. Furthermore, bringing the network to equilibrium with cross-linkers may further stabilize the network against continued polarity sorting seen in our simulations.

**Table 2:** Comparison of our model parameters to parameters in other actomyosin simulation work.

Parameter	Our Simulation	Ennomani, et al. 2016 [54]	Mak, et al, 2016 [55]
motor velocity (v)	1 μm s^-1^	0.3 μm s^-1^	0.14 μm s^-1^
rate of motor attachment (p_1_)	10 s^-1^	5 s^-1^	2,40,1000 s^-1^ rates for transitions between states for binding
rate of motor detachment (p_2_)	1 s^-1^	0.05 s^-1^ rate of unbinding for binder proteins, not defined for myosin motors	2, 20, 1000 s^-1^ rates for transitions between states for unbinding
motor stiffness (k)	3 pN/μm	100 pN/μm	1.69 x 10^4^ pN/μm
Search radius of motor (e.g. maximum stretch of motor, r)	0.3 μm	-	-
maximum motor exerted force on a filament (k · r)	0.9 pN	3.65 pN “rupture” force where motor detaches from actin; based on experimental values for myosin VI	5.7 pN stall force for 64 heads per mini-filament. If we assume that the force/head is constant, than to compare to our 4 headed mini-filaments, the max force is 0.7125 pN
myosin spring rest length	0 μm	0.03 μm (approximately the length of the myosin lever arm)	0.042 μm, however their motor is not represented as a single Hookean spring
Number of motors	5,000	up to 2,000	0.1–5% of actin concentration. If we assume there were 1,000 actin filaments of length 1 μm (like our simulation), then the number of actin monomers would be 1.43x10^5^ (actin. If 0.1–5% of that concentration were motors, that would mean 143–7,150 motors. Furthermore, Mak, et al motors have 64 heads compared to our 4 headed motors, so to compare our number of motors to Mak, et al, we would multiply by 16 to achieve 2,288–114,400 of “our” motors.
Number of filaments	1,000	1,200–1,744 (depending on filament length)	25 μM (a concentration of actin monomer) If we assume that 143 monomers make up a 1 μm long filament, then this concentration would lead to 3.5x10^15^ filaments
Filament length (L)	1 μm	0.95–1.25 μm or 1.45–1.75 μm (uniformly distributed)	1–1.3 μm average length (depending on 2D or 3D domain)
Simulation time	10 s	80 s	47–346 s
Time step size	10 ms	10 ms	0.023 ms
Viscosity (η)	1 pN s/μm^2^	0.18 pN s/μm^2^ in-vitro value with methylcellulose	0.0086 pN s/μm^2^

The recent paper by Ennomani et al [54] also simulated motors as Hookean springs to investigate the formation and stabilization of F-actin rings similar to those that form during cytokinesis. Their simulations operated on initially stabilized actomyosin rings and revealed that actin filaments must be branched or ordered by F-actin cross-linking proteins before contraction. By contrast, in our model ring-like contractile structures emerged as transient features during aster formation. Rings occur as the initial wave of contraction sweeps filaments inward from the boundary. Rings form as inward movement of filaments is initially faster than polarity sorting but occurs without pre-patterning filament alignment or polarity. We also found that addition of actin cross-linking proteins did not stabilize transient rings and only prevented aster formation only when present in excess. We suspected that our simulations differed in the implementation of motor function, since we found motors were capable of driving polarity sorting in filament arrays even when those arrays are cross-linked.

Since Ennomani et al. implemented myosin motors in a manner similar to our implementation, we can directly compare parameters. We note that their motors are stiffer than ours (100 pN/μm versus 3 pN/μm; Table 2), with a rupture force, the point where motors would fall off of a filament, of 3.65 pN compared to our max motor exerted force of 0.9 pN. Additionally, our simulations involve more motors (5,000 compared to Ennomani et al.’s 2,000 motors) that bind F-actin more frequently (10 s^-1^ versus Ennomani et al.’s 5 s^-1^) and move more quickly (1 μm/s versus Ennomani et al.’s 0.3 μm/s). Each myosin motor implemented in our model carry out more work in remodeling filament arrays than those in Ennomani et al., even though the maximum force exerted by our motors is four times less.

Lastly, there is a seemingly large difference in the viscosity between our model and the other two recent actomyosin models. In part, this difference arises from the difference between effective viscosity and dynamic viscosity. Mak et al.’s value is closer to that of water and is included in their calculations for extensional, bending, repulsive and thermal forces for the Brownian motion of filaments, motors, and actin binding proteins. Ennomani et al.’s value is higher and closer to our value (0.18 pN s/μm^2^ versus our value of 1 pN s/μm^2^), but it is not immediately obvious how viscosity in this model influences of filament motion beyond the description that “actin filaments are modeled as elastic fibers surrounded by an immobile viscous fluid” [54]. Viscosity in our model directly regulates the motion of filaments in response to forces exerted by the motors; filament rotation and translation are opposed by viscous drag on a rigid, 1 μm cylindrical rod with a diameter of 8 nm and is based on low Reynolds number hydrodynamics [101].

More detailed models of motor-filament interactions may be more realistic but the increased complexity required to simulate more realistic interactions is not always necessary to demonstrate complex emergent behaviors of actomyosin arrays in the cell cortex. The simplicity of our approach complements existing actomyosin modeling efforts and highlights the strength of microstructural computational methods in exploring the role of F-actin and myosin in shaping the complex mechanics that control cell- and tissue-mechanics and morphogenesis.

## Supporting information

S1 Text Quantifying aster stability. (DOCX)

S2 Text Anchoring filaments and motors. (DOCX)

S3 Text 1D motor-filament model. (DOCX)

S4 Text Quantifying work-energy. (DOCX)

S1 Table Model parameters. (DOCX)

S2 Table Further quantitation of asters in Figs 4 and 5. (DOCX)

S3 Table Quantitation of "No-Aster" cases beyond 1000 time steps. (DOCX)

S1 Fig Distribution plots of the mean motor generated force for the 100 simulations of the sparse filament network. The distributions are normal distributions and have been plotted on the same axes with the exception of the force plot for T = 0 where the motors are all not exerting force on the filaments yet. The mean force at T = 1000 is 0.0935 nN +/- 0.0337 nN.
(TIF)

S2 Fig Mean motor generated force for 10 standard parameter cases. Between Figs 3 and 5 we ran 10 different examples of our standard parameter set. This plot shows the mean motor generated force for those 10 simulations.
(TIF)

S3 Fig Coarse-grained image analysis quantification of asters. We used the coarse-grained image analysis technique to quantify the actin aster evolution from simulated time-lapse sequences (S4 Video), and for the smoothed divergence images (S12 Video). “Actin with Hexs Highlighted” shows where hexagons with mean intensity 1.7-fold higher than the mean intensity over all time for the whole simulation boundary (S4 Video). For the same simulation, we calculated the divergence of the filaments and applied a 2D Gaussian smoothing filter to amplify areas of high/low divergence (S12 Video). We then applied the hexagon intensity map to the divergence data and created a hexagon mask. Once we have highlighted areas identified, we can plot the number of highlighted areas (if more than one hexagon are linked together, they count as one highlighted area; orange), the mean intensity of highlighted areas (again, if more than one hexagon are linked, we calculate the mean intensity within the connected region; purple), area of the highlighted hexagons (blue), and calculated the minimum distance to the boundary for each highlighted area (green). Note: 100 time steps (T) equals 1 second model time.
(TIF)

S4 Fig Color coded orientations of filaments. Filaments are color coded according to their orientation with the plus-end half of the filament shown in green, and the minus end half of the filament shown in red. This example is for no filament turn over (p_2_ = 0).
(TIF)

S5 Fig Extended time analysis for the standard parameter set to T = 3000 time steps. (A) The mean motor generated force for a simulation with the standard parameter set shows a leveling off, or steady state, is reached by 1000 time steps, and remains steady for the duration of the 3000 time step simulation. (B) A kymograph shows that actin filaments quickly condense to the center of the domain to form the aster, and then the aster moves to the side a little bit but stays as an aster.
(TIF)

S6 Fig Tracking filament plus-end recruitment into asters for motor stretch parameter, *r*. The percentage of filament plus-ends that are recruited into the steady state aster for motor stretch threshold value of r = 0.15 and r = 0.6.
(TIF)

S7 Fig Divergence map accompanying Fig 6. Divergence of filaments are shown on the heat map instead of plotting filament locations (Fig 6). Asters are located where the divergence is highlighted by a small cluster of dark pixels with neighboring light pixels. Panel (A) shows how a parameter switch from control to a higher or lower parameter disrupts the aster divergence only in the case of decreasing the chances of motors attaching to filaments (p_1_) or increasing the filament “noise” or turn over (p_2_). (B) Aster morphology can be rescued in all cases except for when filament length is increased to 1 where we observe 2 asters instead of a single aster.
(TIF)

S8 Fig 10 different simulations for parameter switching of L = 0.5 μm to L = 1 μm (standard value). The first row is the morphology of filaments at T = 1000 of the shorter filament length of L = 0.5 μm. The initial orientation and distribution of filaments is random at T = 0. At T = 1001, the filament length has changed to 1 μm and plus-ends remain where they were at T = 1000. The end morphology at T = 2000 is shown in the second row.
(TIF)

S9 Fig Anchored or localized actin filaments remodel and serve as attractors for asters in adjacent regions. (A) Time-evolution of asters under conditions where 10% of 1,000 filaments are fixed in place through their plus-ends. 100 filaments anchored to the bottom fourth of the hexagon (white) and 900 filaments are left free in the domain (red). Over time, the fixed filaments transport motors and trap them. This domain depletes motors from the adjacent regions and allow multiple smaller asters to form. (B) A central 0.5 μm vertical stripe of anchored 100 filaments with 900 free filaments initially form multiple asters. Motors accumulated in the central stripe attract these free asters to the center of the anchored filaments.
(TIF)

S10 Fig Varying the percentage of cross-linkers and motors. Plots of filaments, cross linkers, and motors for simulations where the percentage of total motors (5,000) are designated as cross linkers. Aster formation is inhibited for 75% cross-linkers and 25% motors.
(TIF)

S11 Fig Supporting 1D linear model. (A) Diagram showing the geometry of the model. Motors are filled circles and filaments are lines. Motor 1 will pull filaments a, b, c toward it and those filaments will also pull motor 1. Filament b is attached to two motors. Motor 3 is free to diffuse since it is not bound to any filament. (B) Density for *q* = 0, *r* = 1 and spring constant, *K* = 0.15 showing a single density peak in the three quantities; (C) *q* = 2, *r* = 3 leading to localized interactions with *K* = 0.7 leading a two-peaked density.
(TIF)

S12 Fig Plots of the mean motor generated force over time for varying rates of filament turn over (p_2_). The end motor force decreases as polymerization rate decreases, the same conclusion (and the same shape of the force curve) as we found previously with our simple, 1D rotational model. However, there does seem to be a transition state between p_2_ = 0.3 and p_2_ = 0.7 given the “switch” in the expected maximum force in the beginning of the simulation and the higher than expected steady state force at the end of the simulation.
(TIF)

S13 Fig The organization of filaments and motors for increasing rates of filament turnover. When we look at the organization of filaments (red) and motors (green) at the end of the simulation (t = 10s), we see that motors are localized at the center of filament asters. Additionally, as the rate of filament turnover (p2) increases, motor localization loosens up to eventually form a ring morphology when motor location is projected over the last 5 s of the simulation.
(TIF)

S14 Fig Work-energy calculations over the course of simulations run at four conditions: Standard, high turnover, no turnover, and short filaments. Distinct quasi-static states are observed for each condition: For standard parameters ~ 33% motors are attached and maintain moderate low levels of potential elastic energy. Viscous losses are moderately low. For the high turnover condition similar numbers of motors, ~ 33%, are attached but maintain high levels of potential elastic energy. Viscous losses are high and continuously maintained. For low turnover conditions, few motors are attached, ~ 10%, and maintain very low levels of potential elastic energy and contribute to nearly zero viscous losses. For simulations run with short filaments, fewer motors than the standard case remain attached to filaments with these maintaining low potential elastic energy and low viscous dissipation.
(TIF)

S15 Fig Determining if asters eventually emerge from T = 1000 cases where no aster was evident. We identified cases where simulations did not end in a central aster from Figs 4 to 5 (marked with an *) and ran those simulations for longer times (T = 3000 time steps) to determine if those parameter sets merely delayed aster formation. We have noticed that two simulations ended in asters on the side (η = 5 and r = 0.15), two simulations have clumping on the periphery (p_2_ = 5 and k = 0.5), and three simulations don’t have any evidence of an aster (r = 0.05, p_1_ = 1, and η = 10).
(TIF)

S1 Video Accompanies Fig 1A. Time-lapse sequence of F-actin in the apical (left) and basal (right) cortex of cells within a *Xenopus laevis* animal cap explant at gastrula stage. Video shows differences of actin organization from the same tissue at the same time.
(AVI)

S2 Video Accompanies Fig 1B. Time-lapse sequence of F-actin and myosin II in the basal cell cortex of *Xenopus laevis* dorsal mesoderm at gastrula stages. Video shows episodic actomyosin contractions.
(AVI)

S3 Video Accompanies Fig 3A. Simulated time-lapse sequence of filaments and motors for the standard parameter set.
(AVI)

S4 Video Accompanies Fig 3G. The same video as S3 Video except that it shows the highlighted hexagons from the custom coarse-grained image analysis.
(AVI)

S5 Video Accompanies Fig 4. Simulated time-lapse sequence of filaments and motors for L = 0.25 μm.
(AVI)

S6 Video Accompanies Fig 6. Simulated time-lapse sequence of filaments and motors for parameter switching from L = 0.5 μm to L = 1 μm. Filament lengths for the first half of the movie are short then become longer for the last half of the simulation.
(AVI)

S7 Video Accompanies Fig 7A. Simulated time-lapse sequence of filaments and motors for fixed actin on a bar.
(AVI)

S8 Video Accompanies Fig 7B. Simulated time-lapse sequence of filaments and motors for tethered motors.
(AVI)

S9 Video Accompanies Fig 8. Simulated time-lapse sequence of filaments and motors including cross-linkers.
(AVI)

S10 Video Accompanies Fig 8A. Simulated time-lapse sequence of filaments and motors where filaments are fixed to lower region of the domain.
(AVI)

S11 Video Accompanies Fig 8B. Simulated time-lapse sequence of filaments and motors where filaments are fixed to a central band of the domain.
(AVI)

S12 Video Accompanies S13 Fig. Vertical simulated time-lapse sequences of filaments, motors, and merged images of filaments and motors for standard filament turn over (p2 = 0.7), moderate turn over (p2 = 1.2) and high turnover (p2 = 5).
(AVI)

S13 Video Accompanies S3 Fig. Side by side time-lapse sequences of smoothed divergence mapping for the standard simulation from Fig 3 with the hexagon mask created from the coarse-grained image analysis approach.
(AVI)
