# Supplementary material for: Emergent mechanics of actomyosin drive punctuated contractions and shape network morphology in the cell cortex
Source: PLoS Comput Biol. 2018 Sep 17;14(9):e1006344. doi: 10.1371/journal.pcbi.1006344 (PMC6171965; doi:10.1371/journal.pcbi.1006344)
Supplement: S1 Text — (DOCX) [file pcbi.1006344.s001.docx]

***S1 Text. Quantifying Aster Stability****:*

We use a number of criteria that can be used to quantify the transition of random filament arrays into quasi-stable asters seen in synthetic time-lapse sequences (fig 3A) including kymographs, mean motor generated force, filament divergence, etc. (fig 3 B to G). Each of these methods can reveal rapid evolution of a large central aster in the first few seconds of the simulation and how these large asters sustain a dynamic equilibrium after ~ 7.5 seconds. We describe in this section development of a hybrid approach applying image analysis tools to both image and divergence maps to better quantify the emergence and stability of asters (S3 figure).

***Image Analysis Techniques for Time-lapses:***

To identify regions enriched within F-actin we use a coarse grained image analysis approach to identify small hexagonal regions of high actin intensity. We categorize aster-containing regions that are ~ 2 standard deviations (~ 1.6-fold brighter) above the mean intensity of the larger domain. Once categorized these small hexagonal domains can be clustered and their motion tracked using standard particle tracking methods such as ImageJ Analyze Particles.

Divergence maps can be analyzed using a similar coarse grained approach utilizing a time-lapse sequence of divergence. From a time-lapse map we apply a 2D Gaussian smoothing filter and identify high divergence regions in a 10 x 10 grid based on a standard deviation of $\sigma=2$.

Elements of the filter are calculated as follows:

$f_{i,j}=e^{\frac{-x_{i,j}^{2}}{2\sigma^{2}}-\frac{-y_{i,j}^{2}}{2\sigma^{2}}}$ (8)

$F_{i,j}=\frac{f_{i,j}}{\sum_{i} \sum_{j} f_{i,j}}$ (9)

We can then perform a two-dimensional convolution of the divergence matrix with the 2D Gaussian smoothing filter and use the color heat map to create a movie of the standard simulation case from Figure 3 in the main text.

Once we generated a hexagonal mask for images, we used ImageJ Analyze Particles function to determine centers of the highlighted regions, areas, perimeter, number of separate regions, and created our own macro to determine what the mean intensity was within the highlighted regions. To determine the minimum distance to the boundary, we calculated the distance from the center of a highlighted region to each of the six edges of the domain hexagon. We then took the minimum of those six measurements as a metric for how close highlighted areas are to the outside of the domain.

Both divergence and actin intensity can reveal aster assembly. Actin intensity would be most relevant in comparing models to experiments since detecting F-actin polarity is not yet available to produce *in vivo* divergence maps. For instance, actin intensity can quantify emergence of nascent asters (fig 3G left panel). Divergence hexagonal analysis does not reveal such changes in the filament arrays but can precisely locate asters by the distinctive organization of their cores (fig 3F).

Using the divergence plot, we see a transient stable state most obviously in the calculation of the area of the highlighted hexagons over time (S3 fig). This plot reports the large highlighted area from the transient ring transitioning into the central aster, and leveling off after 600 time steps. Interestingly, we see the transient periphery hexagons begin to populate the domain between 600 to 800 time steps with the small area hexagons on the plot.

Divergence hexagonal analysis of these transient peripheral hexagons enable insights into the spatial pattern of aster emergence and how the central aster might inhibit formation of smaller adjacent asters. As discussed in the main text, smaller F-actin densities form at the periphery and appear to merge with the central aster. These events are easily tracked and revealed by the minimum distance of an aster-hex to the boundary (measured in pixels). This criteria can identify small transient peripheral structures in addition to a large central aster. Using this criteria we see a bifurcation, indicating both a large central aster and transient peripheral structures starting around 600 time steps, after a single central aster has already formed (see minimum distance plot in S3 fig). If we watch the time-lapse, we don’t see these periphery hexagons move into the center, instead they flash and disappear quickly but are clearly resolved in the minimum distance plot.

***Image Analysis Technique for Single Images***

We utilized the coarse-grained image analysis technique for f-actin intensity for single images in figs 4 and 5 to identify the number, area, and distance to the boundary of highlighted areas (see S2 Table). Additionally, we used a divergence map for fig 6 (S7 Fig) to demonstrate another method of determining if asters were present at one point in time.

***Detecting Slow-forming Asters***

Several conditions in figs 4 and 5 do not assemble an aster within 1000 time steps. Since it remained a formal possibility these simulations might generate asters over longer time periods we ran simulations to 3000 time steps in 7 cases (see S3 Table). None of these cases evolved into a large central aster. We used the minimum distance plot to identify two cases where an aster forms on the side of the domain, two cases of clumping or clustering akin to the formation of transient ring-like structures, and three cases where asters never form (two of these last three cases were run to 6000 time steps, S3 Table).
