# Supplementary material for: Emergent mechanics of actomyosin drive punctuated contractions and shape network morphology in the cell cortex
Source: PLoS Comput Biol. 2018 Sep 17;14(9):e1006344. doi: 10.1371/journal.pcbi.1006344 (PMC6171965; doi:10.1371/journal.pcbi.1006344)
Supplement: S2 Text — (DOCX) [file pcbi.1006344.s002.docx]

***S2 Text. Anchoring filaments and motors:***

F-actin stability may also play a role in the formation of actomyosin arrays. To test this possibility, we immobilized a small percentage of free filaments to a specific subdomain and investigated the recruitment of asters into the domain. We tethered 10% of the filament population (100 filaments of the 1,000 filaments total) to a domain in the lower fourth of the hexagon (fig S8A, S9 Movie) yet allowed free motors to bind tethered filaments and direct forces through linkages to free filaments. Interestingly, fixed filaments disrupted formation of the isotropic motor ring structure and segregated motors into multiple asters. A significant fraction of the motors are localized and transport into the densest region of tethered filament plus-ends (fig S9A). When plus-ends were immobilized to the bottom fourth of the hexagonal domain, minus ends of the fixed filaments would rearrange toward the center of the domain enabling free motors to connect the nascent aster to fixed boundary filaments and draw the nascent aster towards the bound domain.

Scaffolds which bind F-actin and hold filaments in a fixed polarity might also control actomyosin formation (Farina et al., 2015). To test whether the orientation of tethered filaments might play a role in aster formation. We positioned filaments with plus-ends pointed toward the center of the hexagon as a way to test if fixed filaments could traffic motors and the filament aster. We found the free filaments congregate into multiple asters that are pulled into the center, following the orientation of the tethered filaments, and eventually assemble into two asters (fig S9B, S10 Movie).

We chose for 10% of actin filaments to be fixed in place (fig S9) and to tether 12.5% of myosin motors (fig 7) based on simplifying assumptions from the series of experiments by Reymann, et al (Reymann, 2012). For the micropatterned experiments, the authors used a concentration of 0.5 μM pWA and 2 μM actin monomers. If we assumed that all pWA bound to the pattern, and actin monomers bound to pWA 1-1, then that would mean that 25% of actin would be fixed to the micropatterend bar. Therefore, we first assumed that any portion of fixed myosin or actin must be less than 25%. If we assume that half of a myosin would be bound to the substrate and the other half would be able to interact with filaments, then for our simulation 12.5% (half of 25%) would be tethered within the domain. In selecting the numbers of fixed actin filaments, we chose 10% because this would mean 100 filaments were fixed.

**Supplementary References:**

**Farina, F., Gaillard, J., Guérin, C., Couté, Y., Sillibourne, J., Blanchoin, L. and Théry, M.** (2015). The centrosome is an actin-organizing centre. *Nature cell biology*.

**Reymann, A.-C., Boujemaa-Paterski, R., Martiel, J.-L., Guerin, C.,Cao, W., Chin, H., De La Cruz, E. M., Thery, M., and Blanchoin, L.** (2012). Actin network architecture can determine myosin motor activity. *Science* **336**, 1310-1314.
