# Supplementary material for: Emergent mechanics of actomyosin drive punctuated contractions and shape network morphology in the cell cortex
Source: PLoS Comput Biol. 2018 Sep 17;14(9):e1006344. doi: 10.1371/journal.pcbi.1006344 (PMC6171965; doi:10.1371/journal.pcbi.1006344)
Supplement: S3 Text — (DOCX) [file pcbi.1006344.s003.docx]

***S3 Text. 1D Model:***

Here we present a simple 1-dimensional model that demonstrates the transition from uniform filament/motor density to clumping into one or more clusters that we take to be analogous to 2D aster formation. The model is one-dimensional in that we examine the density of filaments and motors on a one-dimensional spatial domain so there is no orientation, nor is there motion of the motors along the filaments. Furthermore, to avoid boundary effects and simplify the math, we assume a periodic domain (fig S11A). The model could easily be extended to include more aspects of the 2D simulations but here is presented in minimal form with three variables $f(x,t)$, $\rho_{u}(x,t)$, $\rho_{b}(x,t)$ representing the density of filaments, motors that are bound to filaments, and unbound, free motors. We assume the following interactions and forces:

1. Filaments spontaneously depolymerize and repolymerize at a different location, $k_{1}$;
2. A spring force, $k_{2}(y-x)$ that moves filaments at $x$ toward bound motors at $y$. This effect includes the viscous drag of the filaments (which will be different from the drag of the motors) and also (see below) can be restricted in the distance over which it acts. This and k_5 (y-x) are odd functions as they act like spring forces.
3. Unbound motors at $x$ attach to filaments at $y$ (and thus pull them) at a rate, $k_{3}(x-y)$ and become bound motors. This kernel does not necessarily span the whole domain and has a restricted reach or interaction distance. It is a symmetric function.
4. Bound motors fall off spontaneously, $k_{4}$;
5. The reciprocal spring force of a filament at $y$ pulling a bound motor at $x$, $k_{5}(y-x)$. Similar to k_2 (y-x) this kernel also incorporates the drag of the motors as they are pulled to the filaments and also, by restricting its interaction distance through the parameter q (see below), can incorporate shielding.
6. Unbound motors diffuse at a rate $D$.

Thus, filaments are brought together indirectly through being dragged by the motors. This simplifies the number of variables and the interactions, but, as noted, more complex and realistic models are possible. With these six rates, we obtain the following equations:

$\frac{\partial f(x,t)}{\partial t}=-k_{1}f\left( x,t \right)+\frac{k_{1}}{2\pi}\int_{0}^{2\pi} f\left( y,t \right)dy- \frac{\partial}{\partial x}\left( f(x,t)\int_{0}^{2\pi} k_{2}\left( y-x \right)\rho_{b}\left( y,t \right)dy \right)$ (1)

$\frac{\partial\rho_{u}(x,t)}{\partial t}=D\frac{\partial^{2}\rho_{u}(x,t)}{\partial x^{2}}+k_{4}\rho_{b}\left( x,t \right)-\rho_{u}(x,t)\int_{0}^{2\pi} k_{3}\left( x-y \right)f\left( y,t \right)dy$ (2)

$\frac{\partial\rho_{b}(x,t)}{\partial t}=-k_{4}\rho_{b}\left( x,t \right)+\rho_{u}(x,t)\int_{0}^{2\pi} k_{3}\left( x-y \right)f(y,t)dy- \frac{\partial}{\partial x}\left( \rho_{b}(x,t)\int_{0}^{2\pi} k_{5}\left( y-x \right)f\left( y,t \right)dy \right)$ (3)

The first two terms in eq (1) describe filaments turnover (loss via depolymerization and gain via polymerization) and the third term described motion of filaments driven by bound motors. Bound motors tend to pull the filaments toward them in a manner that depends on their density.

The first term in Eq (2) describes motor diffusion, the second, spontaneous unbinding of motors, and the third, binding of motors to filaments.

The first two terms in Eq (3) describe unbinding and binding of motors and the last term describes the motion of bound motors due to filaments. Total filaments, $2\pi f_{0}=\int_{0}^{2\pi} f\left( x,t \right)dx$ are conserved as is the total number of motors $2\pi\rho_{0}=\int_{0}^{2\pi} \left( \rho_{u}\left( x,t \right)+\rho_{b}\left( x,t \right) \right)dx$.

Before continuing, we describe the three functions, $k_{2}(x)$, $k_{3}(x)$, $k_{5}(x)$. All three are $2\pi$ periodic with $k_{2,5}(x)$ being odd periodic functions and $k_{3}(x)$, an even periodic function. The spring forces should vanish at 0 and be odd symmetric; for example something like $sin(x)$. In our simulations, we use $k_{2,5}\left( x \right)=K_{2,5}\sin\left( x \right)\cos^{2q} (\frac{x}{2})$ and $k_{3}\left( x \right)=K_{3}\cos^{2r} (\frac{x}{2})$. The simplest case sets $q=0$, $r=1$ which allows the motors to bind with any filament in the whole domain and the spring forces to work over the whole spatial domain. This is the “long filament” case. If we restrict the reach of binding and spring forces, we can set $q>0$ and $r>1$ to model the “short filament” case. The larger are these constants, the shorter is the effective interaction range. We can write these functions in their Fourier series as:

$$k_{2}\left( x \right)=\sum_{n=1}^{\infty} \frac{\beta_{n}}{2i}\left[ e^{inx}-e^{-inx} \right]$$

$$k_{5}\left( x \right)=\sum_{n=1}^{\infty} \frac{\gamma_{n}}{2i}\left[ e^{inx}-e^{-inx} \right]$$

$k_{3}\left( x \right)=\alpha_{0}+\sum_{n=1}^{\infty} \frac{\alpha_{n}}{2}\left[ e^{inx}+e^{-inx} \right]$.

There is a uniform steady state, ${(f}_{0},\bar{\rho_{u}}, \bar{\rho_{b}})$, with

$$\bar{\rho_{u}}=\frac{k_{4}}{k_{4}+2\pi\alpha_{0}f_{0}}\rho_{0}$$

$$\bar{\rho_{b}}=\frac{2\pi\alpha_{0}f_{0}}{k_{4}+2\pi\alpha_{0}f_{0}}\rho_{0}$$

The motor density is $\rho_{0}$ and the filament density is $f_{0}$.

We now study the stability of this steady state by linearizing about this equilibrium:

$\frac{\partial f(x,t)}{\partial t}=-k_{1}f\left( x,t \right)+\frac{k_{1}}{2\pi}\int_{0}^{2\pi} f\left( y,t \right)dy- \frac{\partial}{\partial x}\left( f_{0}\int_{0}^{2\pi} k_{2}\left( y-x \right)\rho_{b}\left( y,t \right)dy \right)$ (4)

$\frac{\partial\rho_{u}(x,t)}{\partial t}=D\frac{\partial^{2}\rho_{u}(x,t)}{\partial x^{2}}+k_{4}\rho_{b}\left( x,t \right)-2\pi\alpha_{0}f_{0}\rho_{u}\left( x,t \right)-\bar{\rho_{u}}\int_{0}^{2\pi} k_{3}\left( x-y \right)f\left( y,t \right)dy$ (5)

$\frac{\partial\rho_{b}(x,t)}{\partial t}=-k_{4}\rho_{b}\left( x,t \right)+2\pi\alpha_{0}f_{0}\rho_{u}\left( x,t \right)+ \bar{\rho_{u}}\int_{0}^{2\pi} k_{3}\left( x-y \right)f\left( y,t \right)dy-\frac{\partial}{\partial x}\left( \bar{\rho_{b}}\int_{0}^{2\pi} k_{5}\left( y-x \right)f\left( y,t \right)dy \right)$. (6)

We write $\left( f,\rho_{u}, \rho_{b} \right)=\left( \hat{f},\hat{\rho_{u}},\hat{\rho_{b}} \right)e^{imx}e^{\lambda t}$ and obtain a series of 3x3 matrices for each *m* whose eigenvalues determine the stability of the uniform state. For $m=0$ we get:

$$A_{0}=\left( \begin{matrix} 0 & 0 & 0 \\ -2\pi\alpha_{0}\bar{\rho_{u}} & -2\pi\alpha_{0}f_{0} & k_{4} \\ 2\pi\alpha_{0}\bar{\rho_{u}} & 2\pi\alpha_{0}f_{0} & -k_{4} \end{matrix} \right)$$

This is clearly a rank 1 matrix with a single nonzero eigenvalue, $-2\pi\alpha_{0}f_{0}-k_{4}$ which is negative. The two zero eignevalues come from the independent conservation of filaments and motors. For $m>0$, we obtain:

$A_{m}=\left( \begin{matrix} -k_{1} & 0 & f_{0}\beta_{m}m\pi\\ -\bar{\rho_{u}}\pi\alpha_{m} & -Dm^{2}-2\pi\alpha_{0}f_{0} & k_{4} \\ \bar{\rho_{u}}\pi\alpha_{m}+\bar{\rho_{b}}\gamma_{m}m\pi& 2\pi\alpha_{0}f_{0} & -k_{4} \end{matrix} \right)$.

With the help of a computer algebra system, we can compute the characteristic equation of $A_{m}$, $\rho\left( \lambda\right)=\lambda^{3}+C_{2}\lambda^{2}+C_{1}\lambda+C_{0}$. One can then study the signs of the three coefficients as well as the Routh-Hurwitz condition that $C_{1}C_{2}-C_{0}>0$. We find that $C_{2}>0$ unconditionally. We also find that as long as $C_{0}>0$, then so are $C_{1}$ and the Routh Hurwitz condition holds. Thus, the instability of $A_{m}$ occurs when $C_{0}$ changes from positive to negative; that is at a zero eigenvalue. If this happens at some value of $m_{0}$ and $C_{0}>0$ for all other values of *m*, then we obtain a so-called pattern forming instability that will produce $m_{0}$ clusters. We consider two parameters to vary, $\rho_{0}$, the density of motors, and $K_{2}=K_{5}=5$, the maximum spring constant. For each *m*, we can show that there is a classical density ($\rho\_C$ where $C_{0}$ vanishes.

$\rho_{C\left( m \right)}=\frac{k_{1}Dm^{2}k_{4}\left( k_{4}+2\pi\alpha_{0} \right)}{m\beta\_mf\_0(2\pi\alpha_{0}f_{0}\gamma_{m}Dm^{3}+my_{m}\left( 2\pi\alpha_{0}f_{0} \right)2+k_{4}\alpha_{m}Dm^2}$. (7)

The coefficient *C_0_* is quadratic in K, so the critical spring coupling is a complicated expression. However, it is easy to show that there is always exactly one positive root using Descarte’s rule of signs. We run a numerical simulation of this simple model by discretizing space in equations 1-3 into 100 bins. With parameters, $q=0$, $r=1$, $f_{0}=1$, $k_{4}=0.5$, $\alpha_{0}=0.3$, $k_{1}=0.5$, $\rho_{0}=0.2$, we get a critical spring constant at $m=1$, $K=0.12233$. Supplement figure S11 shows two different simulations with $q=0$, $r=1$ (fig S11 B) and with $q=2$, $r=3$ (fig S11 C). In the latter simulation, the reach of the motor-filament interactions is much less than in the former (“short filament”) leading to two clusters.
