# Supplementary material for: Emergent mechanics of actomyosin drive punctuated contractions and shape network morphology in the cell cortex
Source: PLoS Comput Biol. 2018 Sep 17;14(9):e1006344. doi: 10.1371/journal.pcbi.1006344 (PMC6171965; doi:10.1371/journal.pcbi.1006344)
Supplement: S4 Text — (DOCX) [file pcbi.1006344.s004.docx]

***S4 Text. Quantifying Work-Energy****:*

To understand the amount of potential energy stored in the motors and the energy dissipated due to filament drag through the viscous fluid, we first calculated the number of motors (out of the total of 5,000 motors for the entire simulation) in each of the three states: attached (red), unattached (blue) or only one leg attached (green) (see S14 Figure). To determine the potential energy in the motors, we broke the motors into each of the three motor state categories, and determined the amount of potential energy due to the spring and averaged over all motors in each state.

$$Energy=\frac{1}{2}*k*\Delta x^{2}$$

As expected, when motors are unattached, their change in motor stretch ($\Delta x$) is extremely small. The only reason why it would be some number greater than 0 would be that the motor was in the process of falling off.

Next, we wanted to calculate the amount of energy dissipated due to filament drag through the viscous fluid. At each time step, we calculated how filaments moved due to motor exerted force and resistance from moving through a viscous fluid. We then quantified the viscous energy for each filament (*i*) as a sum of forces lost to drag by filament *i* moving in the parallel, perpendicular, and rotational directions:

$$Dissipated Energy_{i}=\sum_{j} {{FR}_{j}(1)}^{2}*\frac{dt}{\Gamma_{par}}+\sum_{j} {{FR}_{j}\left( 2 \right)}^{2}*\frac{dt}{\Gamma_{perp}}+\sum_{j} {{(len_{j}*FR}_{j}(2))}^{2}*\frac{dt}{\Gamma_{rot}}$$

To obtain the total viscous dissipated energy at each time, we sum the viscous energy dissipated for all filaments and then plot the change in energy over time.
