# Supplementary material for: Emergent mechanics of actomyosin drive punctuated contractions and shape network morphology in the cell cortex
Source: PLoS Comput Biol. 2018 Sep 17;14(9):e1006344. doi: 10.1371/journal.pcbi.1006344 (PMC6171965; doi:10.1371/journal.pcbi.1006344)
Supplement: S1 Table — (DOCX) [file pcbi.1006344.s005.docx]

**S1 Table. Model Parameters**

| Parameter | Symbol | Model value | *In vitro* value | Reference |
| --- | --- | --- | --- | --- |
| Simulation  Duration | - | 10 seconds | ~1 to 2 minutes | (von Dassow et al., 2014) |
| Number of filaments | N | 1000 |  |  |
| Number of motors | M | 5000 |  |  |
| Persistence length | L | 1 µm | 6 nm to 10 µm | (Murrell and Gardel, 2012) |
| Rate of motor detachment | p_0_ | 1s^-1^ | Lower than p_1_ |  |
| Rate of motor attachment | p_1_ | 10s^-1^ | Higher than p_0_ |  |
| Rate of filament polymerization | p_2_ | 0.7s^-1^ | 0.7 to 1.2/s | (Amann, 2001; Pollard, 1981) |
| Time step size | h | 0.01 s |  |  |
| Search radius of motor | r | 0.3 µm | ~0.3 µm length of a single NMM II | (Shutova et al., 2012) |
| Motor velocity | v | 1 µm/s | 1 to 3 µm/s | (Kron and Spudich, 1986; Murphy et al., 2001) |
| Dynamic viscosity | η | 1 pN s/µm^2^ | 8.9 x 10^-4^  pN s/µm^2^ | (Hunt, 1994) |
| Motor stiffness | k | 3 pN/µm | 1.875 pN/µm (single myosin) to 1,250 pN/µm (skeletal muscle) | (Dunaway et al., 2002; Kaya and Higuchi, 2010; Nagornyak et al., 2005; Neumann et al., 1998) |
| Rate of motor diffusion | d | 0.02/s |  |  |
| Mean step size of motor diffusion | µ | 0.001 µm |  |  |
| Diameter of actin filament | di | 0.008 µm | 0.006 to 0.008 μm | (Howard, 2001) |
| Domain size (hexagon diameter) | - | 2 µm | 5 µm in 2D simulation | (Dasanayake, 2011) |
| Angle of crosslinking | - | <22.5º | 0 to 90º | (Cooper, 2000) |
| Cross-linker span | - | 40 nm | 10 to 40 nm | (Cooper, 2000; Falzone, 2012; Pollard and Cooper, 1986) |

**Supplementary References:**

**Amann, K. J. a. P., T. D.** (2001). Direct real-time observation of actin ﬁlament branching mediated by arp 2/3 complex using total internal reﬂection ﬂuorescence microscopy. *Proceedings of the National Academy of Sciences of the United States of America*, 15009–15013.

**Cooper, G.** (2000). *The Cell: A Molecular Approach* (2nd edn). Sunderland, MA: Sinauer Associates.

**Dasanayake, N., Michalski, P., and Anders, E.** (2011). General mechanism of actomyosin contractility. *Physical review letters* **107**.

**Dunaway, D., Fauver, M. and Pollack, G.** (2002). Direct measurement of single synthetic vertebrate thick filament elasticity using nanofabricated cantilevers. *Biophys J* **82**, 3128-3133.

**Falzone, T., Lenz, M., Kovar, D., and Gardel, M.** (2012). Assembly kinetics determine the architecture of α-actinin cross linked f-actin networks. *Nature Communications* **3**.

**Farina, F., Gaillard, J., Guérin, C., Couté, Y., Sillibourne, J., Blanchoin, L. and Théry, M.** (2015). The centrosome is an actin-organizing centre. *Nature cell biology*.

**Howard, J.** (2001). *Mechanisms of Motor Proteins and the Cytoskeleton*. Sunderland, MA: Sinauer Associates.

**Hunt, A. J., Gittes, F., and Howard, J.** (1994). The force exerted by a single kinesin molecule against a viscous load. *Biophysical journal* **67**, 766-781.

**Kaya, M. and Higuchi, H.** (2010). Nonlinear elasticity and an 8-nm working stroke of single myosin molecules in myofilaments. *Science* **329**, 686-689.

**Kron, S. J. and Spudich, J. A.** (1986). Fluorescent actin filaments move on myosin fixed to a glass surface. *Proc Natl Acad Sci U S A* **83**, 6272-6276.

**Murphy, C. T., Rock, R. S. and Spudich, J. A.** (2001). A myosin II mutation uncouples ATPase activity from motility and shortens step size. *Nat Cell Biol* **3**, 311-315.

**Murrell, M. P. and Gardel, M. L.** (2012). F-actin buckling coordinates contractility and severing in a biomimetic actomyosin cortex. *Proceedings of the National Academy of Sciences of the United States of America* **109**, 20820-20825.

**Nagornyak, E. M., Blyakhman, F. A. and Pollack, G. H.** (2005). Stepwise length changes in single invertebrate thick filaments. *Biophys J* **89**, 3269-3276.

**Neumann, T., Fauver, M. and Pollack, G. H.** (1998). Elastic properties of isolated thick filaments measured by nanofabricated cantilevers. *Biophys J* **75**, 938-947.

**Pollard, T. D.** (1981). Purification of a calcium-sensitive actin gelation protein from Acanthamoeba. *The Journal of biological chemistry* **256**, 7666-7670.

**Pollard, T. D. and Cooper, J. A.** (1986). Actin and actin-binding proteins. A critical evaluation of mechanisms and functions. *Annual review of biochemistry* **55**, 987-1035.

**Reymann, A.-C., Boujemaa-Paterski, R., Martiel, J.-L., Guerin, C.,Cao, W., Chin, H., De La Cruz, E. M., Thery, M., and Blanchoin, L.** (2012). Actin network architecture can determine myosin motor activity. *Science* **336**, 1310-1314.

**Shutova, M., Yang, C., Vasiliev, J. M. and Svitkina, T.** (2012). Functions of nonmuscle myosin II in assembly of the cellular contractile system. *PloS one* **7**, e40814.

**von Dassow, M., Miller, C. J. and Davidson, L. A.** (2014). Biomechanics and the thermotolerance of development. *PLoS One* **9**, e95670.
