# Supplementary material for: Emergent mechanics of actomyosin drive punctuated contractions and shape network morphology in the cell cortex
Source: PLoS Comput Biol. 2018 Sep 17;14(9):e1006344. doi: 10.1371/journal.pcbi.1006344 (PMC6171965; doi:10.1371/journal.pcbi.1006344)
Supplement: S2 Table — (DOCX) [file pcbi.1006344.s006.docx]

**S2 Table. Further Quantitation of Asters in Figures 4 and 5.**

| **Parameter Name and Value** | **Number of hexagon regions** | **Area of hexagon regions (pixels^2^)** | **Minimum Distance to the Boundary of hexagon regions (pixels)** |
| --- | --- | --- | --- |
| η=0.1 | 2 | 5328 | 18584.93 |
|  |  | 672 | 29913.21 |
| η=0.5 | 2 | 1344 | 27885.54 |
|  |  | 1296 | 25553.25 |
| η=5 | 5 | 672 | 28186.00 |
|  |  | 648 | 13510.21 |
|  |  | 672 | 8146.00 |
|  |  | 1320 | 21123.18 |
|  |  | 672 | 10912.32 |
| η=10 | 0 | n.d.^1^ |  |
| k=0 | 0 | n.d. |  |
| k=0.5 | 2 | 672 | 7615.70 |
|  |  | 648 | 12022.31 |
| k=1 | 4 | 1320 | 9960.38 |
|  |  | 672 | 22816.85 |
|  |  | 672 | 21922.00 |
|  |  | 672 | 16290.00 |
| k=10 | 3 | 672 | 7371.29 |
|  |  | 672 | 32890.67 |
|  |  | 1320 | 27720.27 |
| k=60 | 2 | 19272 | 19825.28 |
|  |  | 672 | 18061.00 |
| L=0.5 | 3 | 672 | 3630.27 |
|  |  | 3312 | 17279.75 |
|  |  | 1968 | 10739.16 |
| L=0.25 | 10 | 1320 | 11255.71 |
|  |  | 1296 | 26832.25 |
|  |  | 1992 | 10326.61 |
|  |  | 1344 | 7632.00 |
|  |  | 1344 | 2809.00 |
|  |  | 1296 | 31192.41 |
|  |  | 1968 | 19131.09 |
|  |  | 1320 | 12835.34 |
|  |  | 672 | 8922.86 |
|  |  | 672 | 2721.86 |
| L=1.5 | 0 | n.d. |  |
| L=2 | 0 | n.d. |  |
| p0=0.25 | 1 | 672 | 36111.49 |
| p0=5 | 1 | 648 | 35432.86 |
| p0=10 | 3 | 1344 | 23704.62 |
|  |  | 648 | 25499.59 |
|  |  | 672 | 25236.00 |
| p1=1 | 0 | n.d. |  |
| p1=5 | 1 | 1344 | 37637.00 |
| p1=20 | 1 | 1320 | 35682.76 |
| p1=30 | 2 | 672 | 16521.21 |
|  |  | 1992 | 31654.91 |
| p2=0 | 3 | 8640 | 38120.67 |
|  |  | 3312 | 13683.87 |
|  |  | 1320 | 17284.48 |
| p2=0.3 | 1 | 1320 | 31213.89 |
| p2=1.2 | 2 | 1344 | 32050.00 |
|  |  | 672 | 28529.16 |
| p2=5 | 0 | n.d. |  |
| r=0.05 | 0 | n.d. |  |
| r=0.15 | 0 | n.d. |  |
| r=0.6 | 2 | 672 | 27425.00 |
|  |  | 7968 | 39183.75 |
| r=1.2 | 1 | 24552 | 36548.26 |
| v=0.25 | 1 | 1320 | 24345.02 |
| v=0.5 | 1 | 648 | 36598.96 |
| v=2 | 1 | 648 | 29852.64 |
| v=3 | 2 | 648 | 17072.66 |
|  |  | 672 | 33653.00 |
| Standard Run 1 | 1 | 648 | 24667.00 |
| Standard Run 2 | 1 | 1344 | 28250.00 |
| Standard Run 3 | 1 | 1944 | 35182.11 |
| Standard Run 4 | 2 | 672 | 28234.23 |
|  |  | 648 | 26204.32 |
| Standard Run 5 | 1 | 672 | 28626.23 |
| Standard Run 6 | 1 | 1344 | 27226.00 |
| Standard Run 7 | 1 | 1992 | 28978.12 |
| Standard Run 8 | 3 | 672 | 32084.85 |
|  |  | 672 | 30706.00 |
|  |  | 648 | 14345.03 |
| Standard Run 9 | 1 | 3984 | 31010.22 |
| Standard Run 10 | 3 | 672 | 12645.73 |
|  |  | 672 | 25796.00 |
|  |  | 1320 | 34152.55 |

^1^ n.d. no data.
