# Supplementary material for: Emergent mechanics of actomyosin drive punctuated contractions and shape network morphology in the cell cortex
Source: PLoS Comput Biol. 2018 Sep 17;14(9):e1006344. doi: 10.1371/journal.pcbi.1006344 (PMC6171965; doi:10.1371/journal.pcbi.1006344)
Supplement: S3 Table — (DOCX) [file pcbi.1006344.s007.docx]

**S3 Table. Quantitation of "No-Aster" Cases Beyond 1000 Time Steps.**

| **Parameter Name and Value** | **Number of hexagon regions (T=3000 time steps)** | **Area of hexagon regions (pixels^2^) (T=3000 time steps)** | **Minimum Distance to the Boundary of hexagon regions (pixels) (T=3000 time steps)** | **Number of hexagon regions (T=6000 time steps)** | **Area of hexagon regions (pixels^2^) (T=6000 time steps)** | **Minimum Distance to the Boundary of hexagon regions (pixels) (T=6000 time steps)** |
| --- | --- | --- | --- | --- | --- | --- |
| η=5 | 2 | 672 | 12469.00 | n.d.^1^ |  |  |
|  |  | 1320 | 20364.82 | n.d. |  |  |
| η=10 | 1 | 648 | 20280.43 | 1 | 672 | 11765.00 |
| k=0.5 | 2 | 648 | 18360.03 | n.d. |  |  |
|  |  | 648 | 13239.08 | n.d. |  |  |
| p1=1 | 1 | 672 | 14487.32 | n.d. |  |  |
| p2=5 | 1 | 672 | 12483.11 | 1 | 648 | 18360.03 |
|  | 2 | 672 | 10440.96 | 2 | 648 | 13239.08 |
|  | 3 | 648 | 18224.89 | n.d. |  |  |
| r=0.05 | 0 |  |  | n.d. |  |  |
| r=0.15 | 1 | 672 | 18412.70 | n.d. |  |  |

^1^ n.d. no data.
