# Supplementary figures and images for: Emergent mechanics of actomyosin drive punctuated contractions and shape network morphology in the cell cortex
Source: PLoS Comput Biol. 2018 Sep 17;14(9):e1006344. doi: 10.1371/journal.pcbi.1006344 (PMC6171965; doi:10.1371/journal.pcbi.1006344)

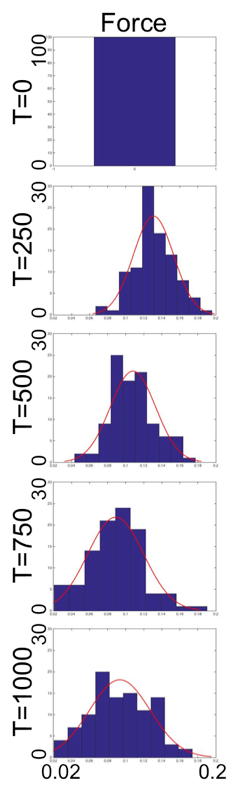

Supplement: S1 Fig — The distributions are normal distributions and have been plotted on the same axes with the exception of the force plot for T = 0 where the motors are all not exerting force on the filaments yet. The mean force at T = 1000 is 0.0935 nN +/- 0.0337 nN. (TIF) [file pcbi.1006344.s008.tif]

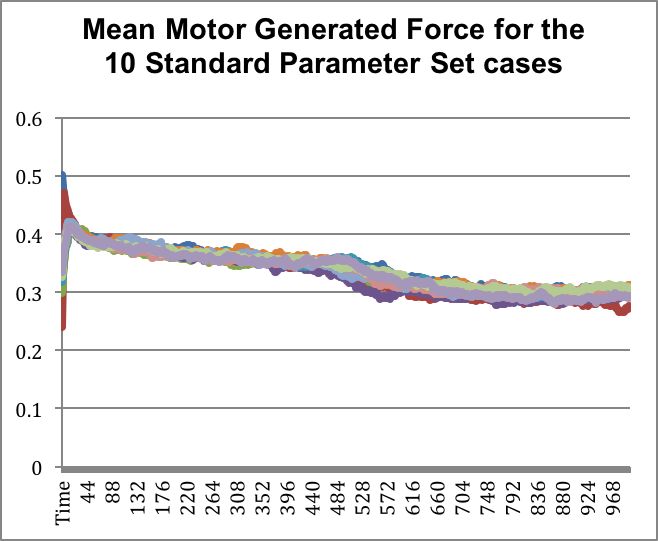

Supplement: S2 Fig — Between Figs 3 and 5 we ran 10 different examples of our standard parameter set. This plot shows the mean motor generated force for those 10 simulations. (TIF) [file pcbi.1006344.s009.tif]

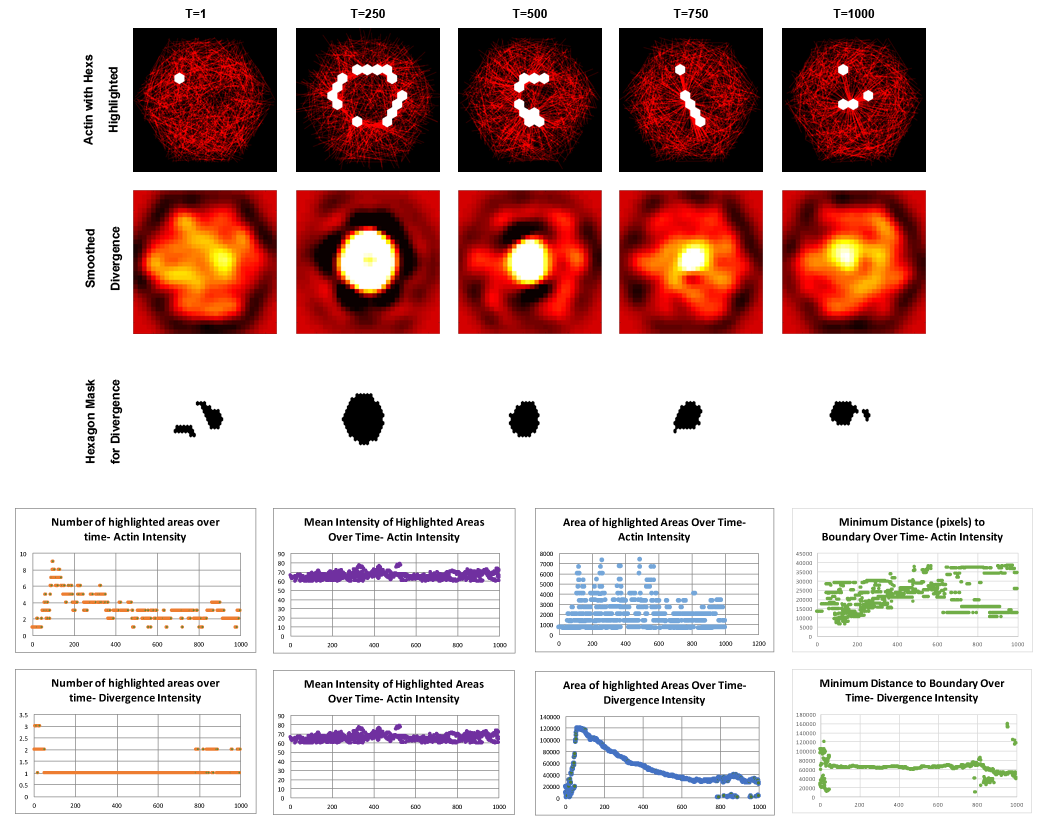

Supplement: S3 Fig — We used the coarse-grained image analysis technique to quantify the actin aster evolution from simulated time-lapse sequences (S4 Video), and for the smoothed divergence images (S12 Video). “Actin with Hexs Highlighted” shows where hexagons with mean intensity 1.7-fold higher than the mean intensity over all time for the whole simulation boundary (S4 Video). For the same simulation, we calculated the divergence of the filaments and applied a 2D Gaussian smoothing filter to amplify areas of high/low divergence (S12 Video). We then applied the hexagon intensity map to the divergence data and created a hexagon mask. Once we have highlighted areas identified, we can plot the number of highlighted areas (if more than one hexagon are linked together, they count as one highlighted area; orange), the mean intensity of highlighted areas (again, if more than one hexagon are linked, we calculate the mean intensity within the connected region; purple), area of the highlighted hexagons (blue), and calculated the minimum distance to the boundary for each highlighted area (green). Note: 100 time steps (T) equals 1 second model time. (TIF) [file pcbi.1006344.s010.tif]

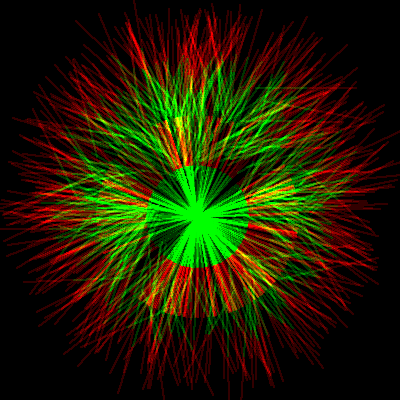

Supplement: S4 Fig — Filaments are color coded according to their orientation with the plus-end half of the filament shown in green, and the minus end half of the filament shown in red. This example is for no filament turn over (p2 = 0). (TIF) [file pcbi.1006344.s011.tif]

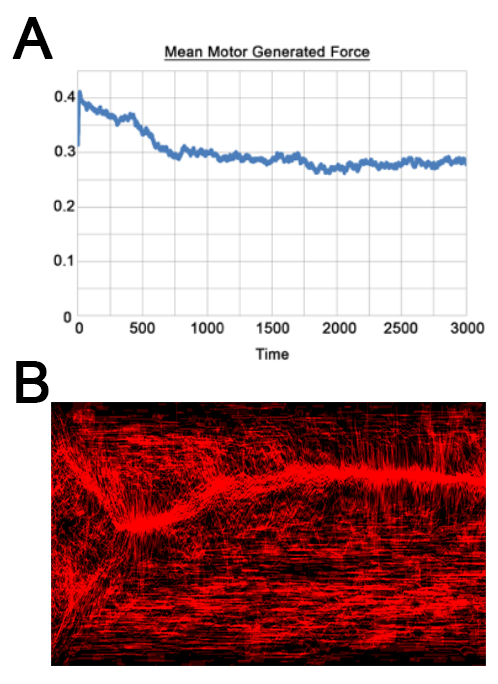

Supplement: S5 Fig — (A) The mean motor generated force for a simulation with the standard parameter set shows a leveling off, or steady state, is reached by 1000 time steps, and remains steady for the duration of the 3000 time step simulation. (B) A kymograph shows that actin filaments quickly condense to the center of the domain to form the aster, and then the aster moves to the side a little bit but stays as an aster. (TIF) [file pcbi.1006344.s012.tif]

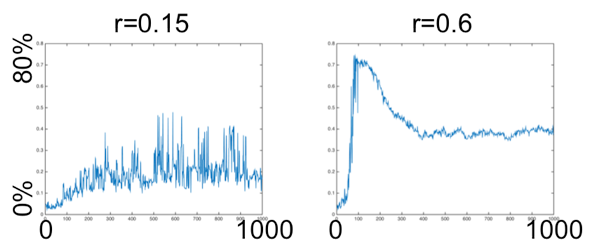

Supplement: S6 Fig — The percentage of filament plus-ends that are recruited into the steady state aster for motor stretch threshold value of r = 0.15 and r = 0.6. (TIF) [file pcbi.1006344.s013.tif]

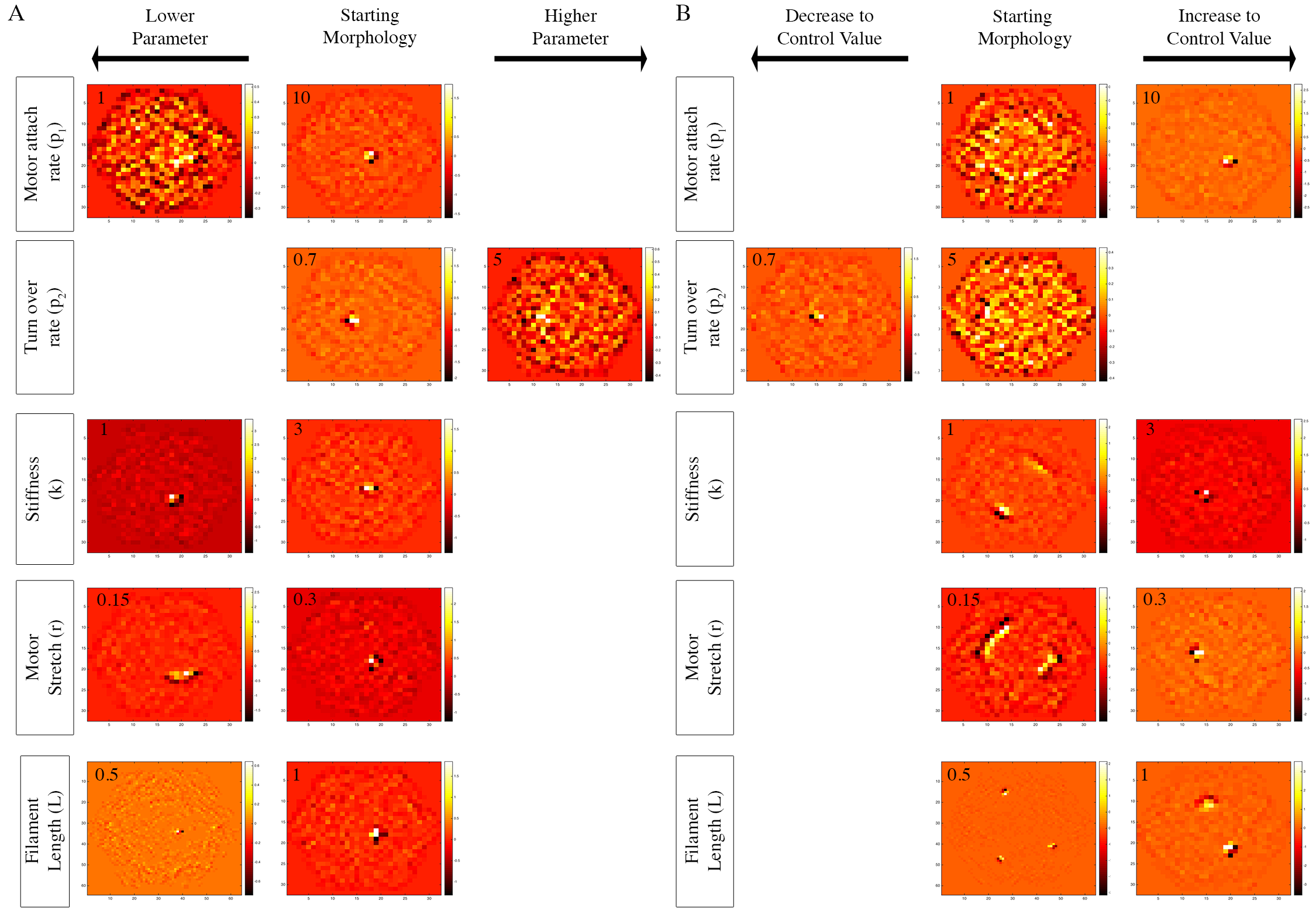

Supplement: S7 Fig — Divergence of filaments are shown on the heat map instead of plotting filament locations (Fig 6). Asters are located where the divergence is highlighted by a small cluster of dark pixels with neighboring light pixels. Panel (A) shows how a parameter switch from control to a higher or lower parameter disrupts the aster divergence only in the case of decreasing the chances of motors attaching to filaments (p1) or increasing the filament “noise” or turn over (p2). (B) Aster morphology can be rescued in all cases except for when filament length is increased to 1 where we observe 2 asters instead of a single aster. (TIF) [file pcbi.1006344.s014.tif]

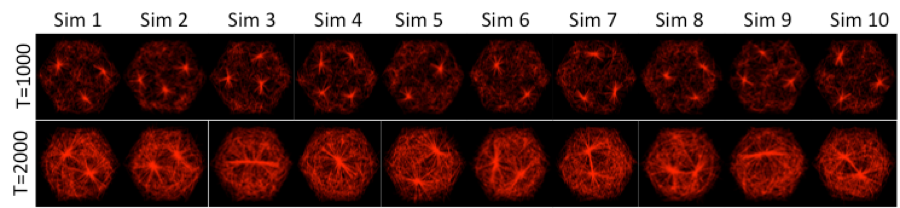

Supplement: S8 Fig — The first row is the morphology of filaments at T = 1000 of the shorter filament length of L = 0.5 μm. The initial orientation and distribution of filaments is random at T = 0. At T = 1001, the filament length has changed to 1 μm and plus-ends remain where they were at T = 1000. The end morphology at T = 2000 is shown in the second row. (TIF) [file pcbi.1006344.s015.tif]

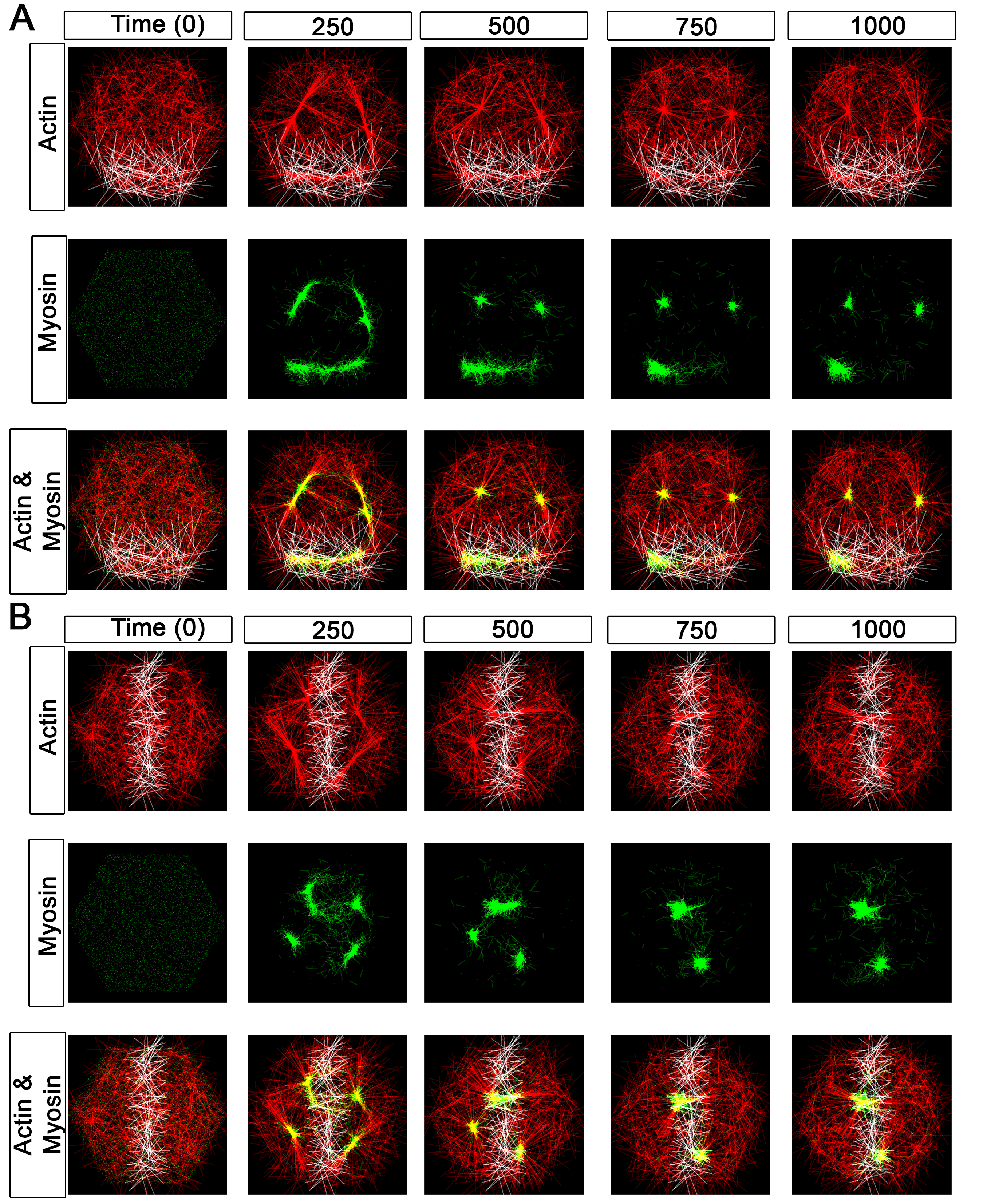

Supplement: S9 Fig — (A) Time-evolution of asters under conditions where 10% of 1,000 filaments are fixed in place through their plus-ends. 100 filaments anchored to the bottom fourth of the hexagon (white) and 900 filaments are left free in the domain (red). Over time, the fixed filaments transport motors and trap them. This domain depletes motors from the adjacent regions and allow multiple smaller asters to form. (B) A central 0.5 μm vertical stripe of anchored 100 filaments with 900 free filaments initially form multiple asters. Motors accumulated in the central stripe attract these free asters to the center of the anchored filaments. (TIF) [file pcbi.1006344.s016.tif]

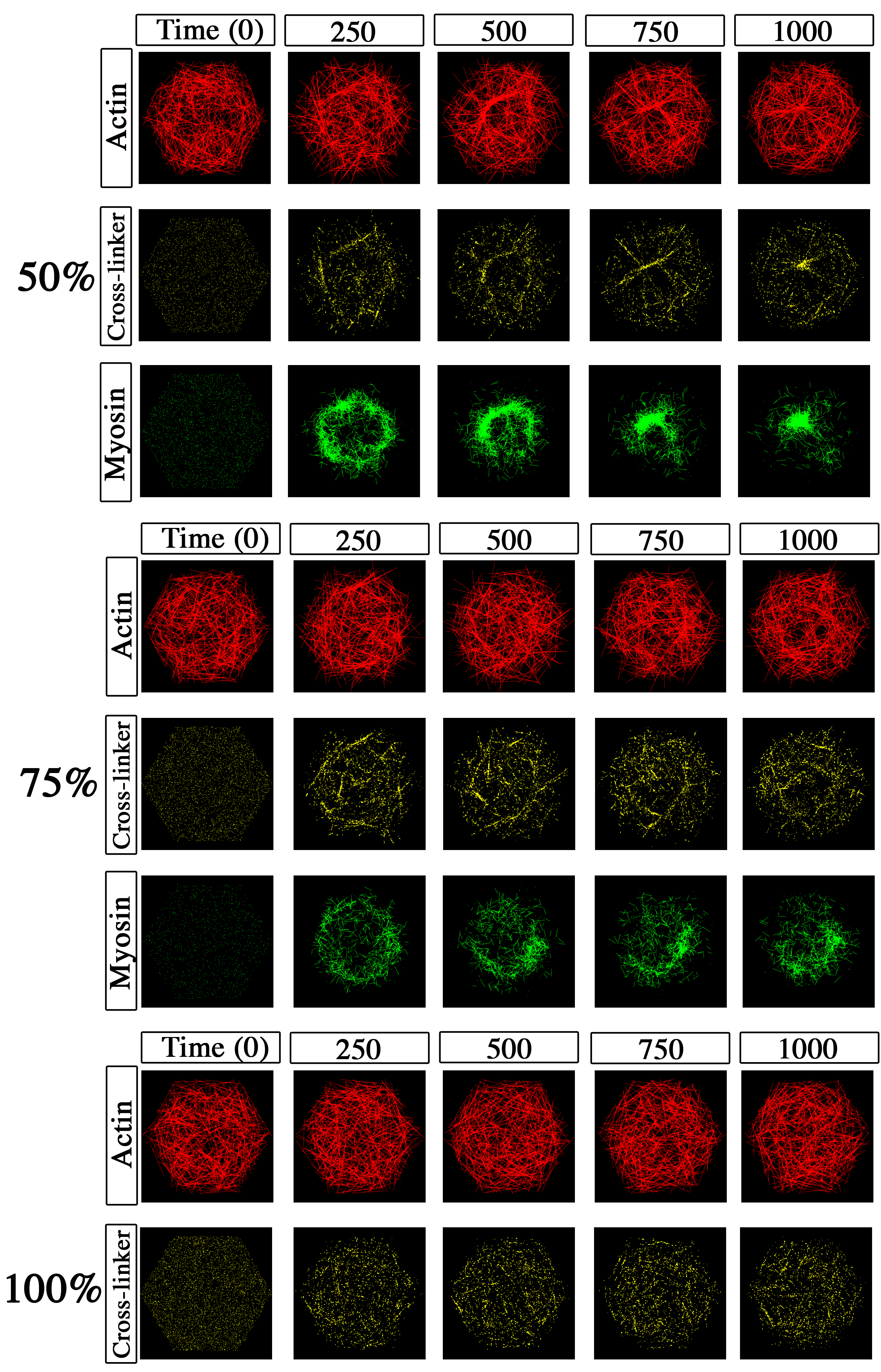

Supplement: S10 Fig — Plots of filaments, cross linkers, and motors for simulations where the percentage of total motors (5,000) are designated as cross linkers. Aster formation is inhibited for 75% cross-linkers and 25% motors. (TIF) [file pcbi.1006344.s017.tif]

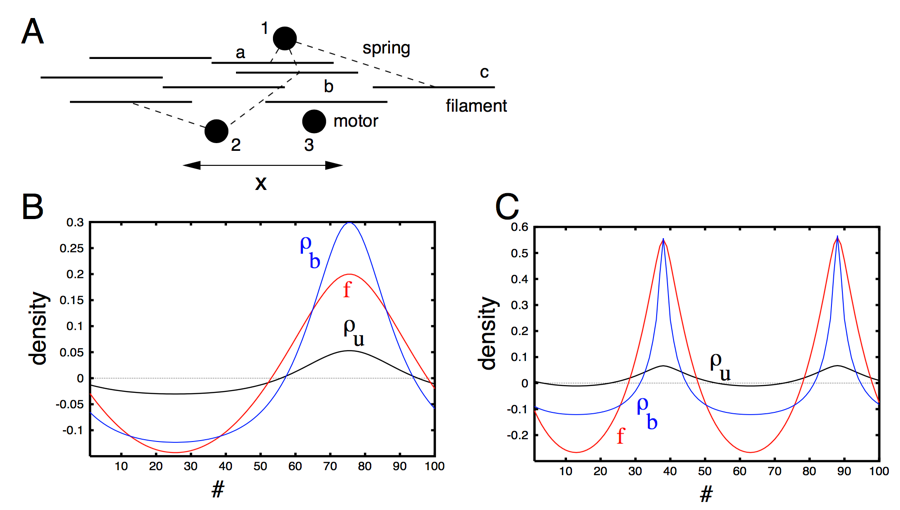

Supplement: S11 Fig — (A) Diagram showing the geometry of the model. Motors are filled circles and filaments are lines. Motor 1 will pull filaments a, b, c toward it and those filaments will also pull motor 1. Filament b is attached to two motors. Motor 3 is free to diffuse since it is not bound to any filament. (B) Density for q = 0, r = 1 and spring constant, K = 0.15 showing a single density peak in the three quantities; (C) q = 2, r = 3 leading to localized interactions with K = 0.7 leading a two-peaked density. (TIF) [file pcbi.1006344.s018.tif]

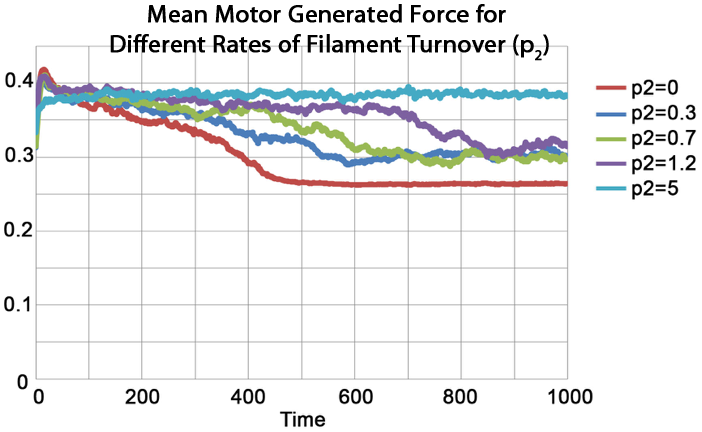

Supplement: S12 Fig — The end motor force decreases as polymerization rate decreases, the same conclusion (and the same shape of the force curve) as we found previously with our simple, 1D rotational model. However, there does seem to be a transition state between p2 = 0.3 and p2 = 0.7 given the “switch” in the expected maximum force in the beginning of the simulation and the higher than expected steady state force at the end of the simulation. (TIF) [file pcbi.1006344.s019.tif]

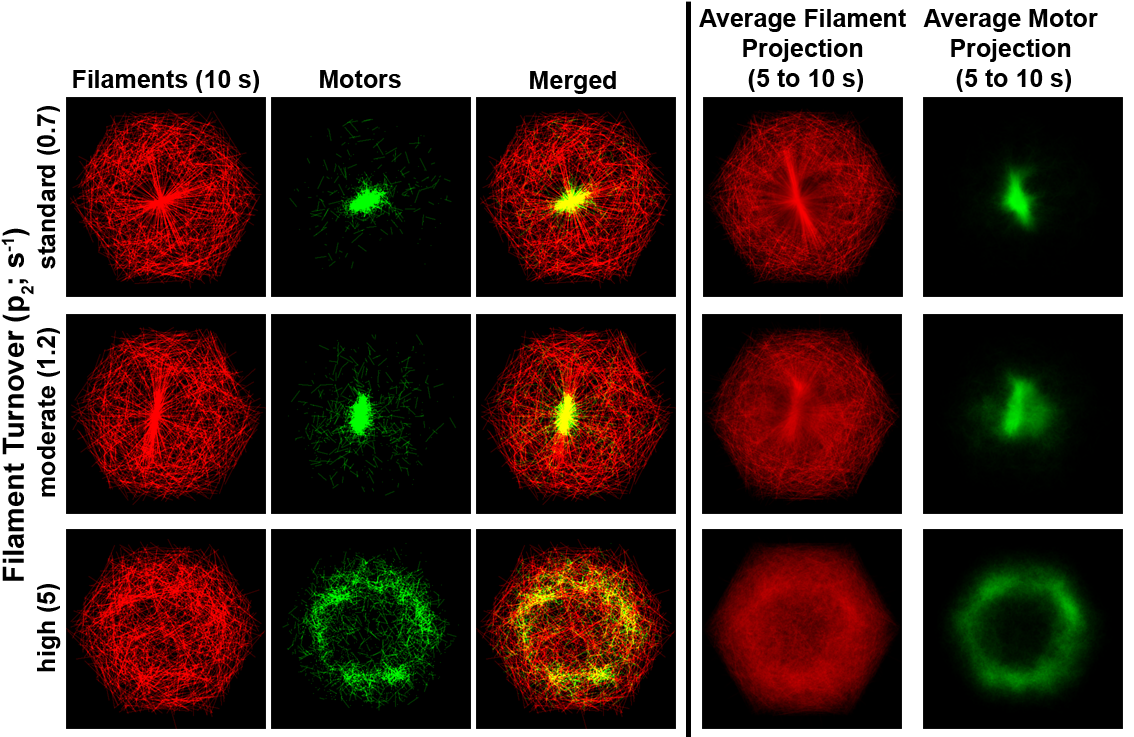

Supplement: S13 Fig — When we look at the organization of filaments (red) and motors (green) at the end of the simulation (t = 10s), we see that motors are localized at the center of filament asters. Additionally, as the rate of filament turnover (p2) increases, motor localization loosens up to eventually form a ring morphology when motor location is projected over the last 5 s of the simulation. (TIF) [file pcbi.1006344.s020.tif]

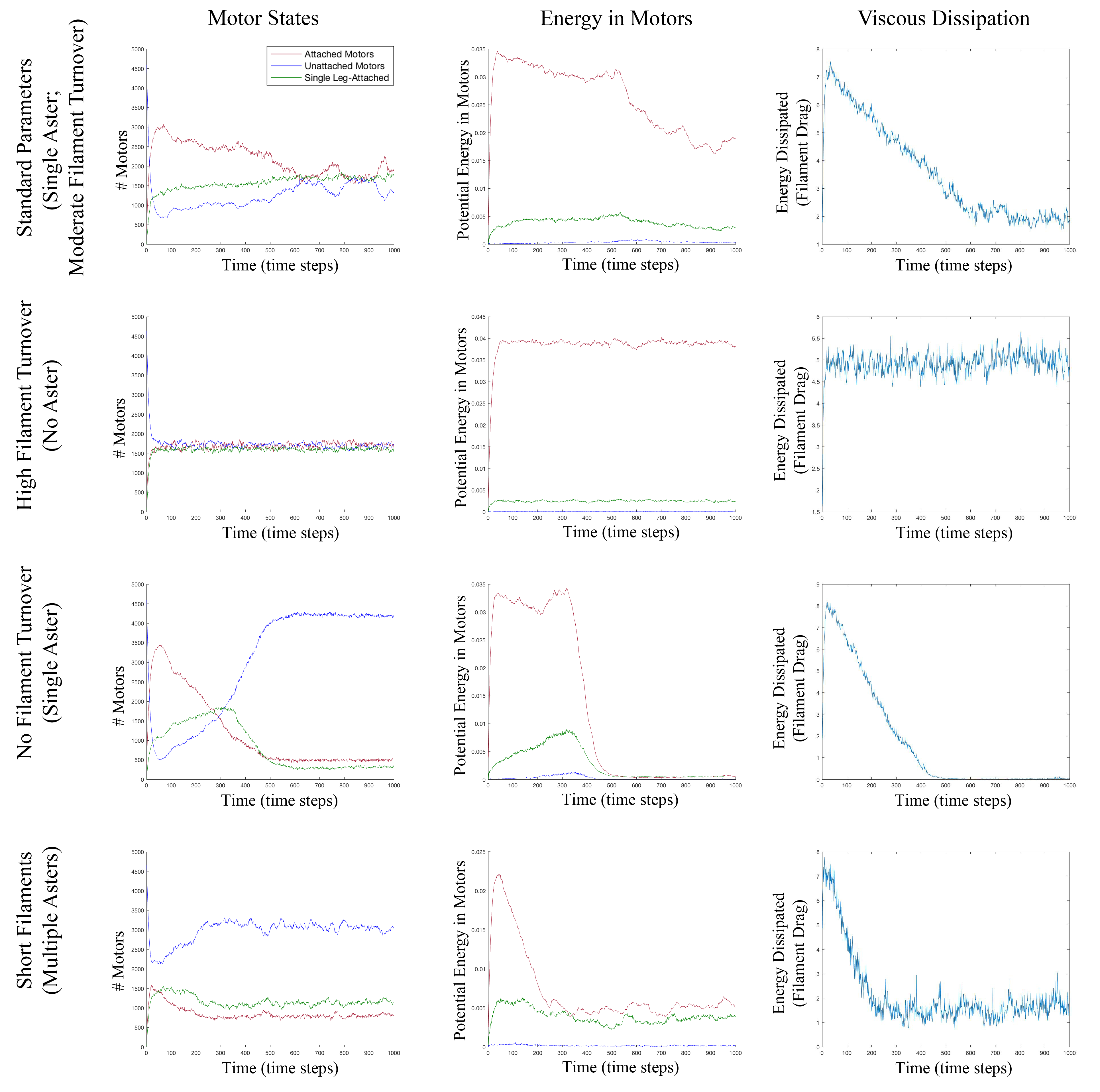

Supplement: S14 Fig — Distinct quasi-static states are observed for each condition: For standard parameters ~ 33% motors are attached and maintain moderate low levels of potential elastic energy. Viscous losses are moderately low. For the high turnover condition similar numbers of motors, ~ 33%, are attached but maintain high levels of potential elastic energy. Viscous losses are high and continuously maintained. For low turnover conditions, few motors are attached, ~ 10%, and maintain very low levels of potential elastic energy and contribute to nearly zero viscous losses. For simulations run with short filaments, fewer motors than the standard case remain attached to filaments with these maintaining low potential elastic energy and low viscous dissipation. (TIF) [file pcbi.1006344.s021.tif]

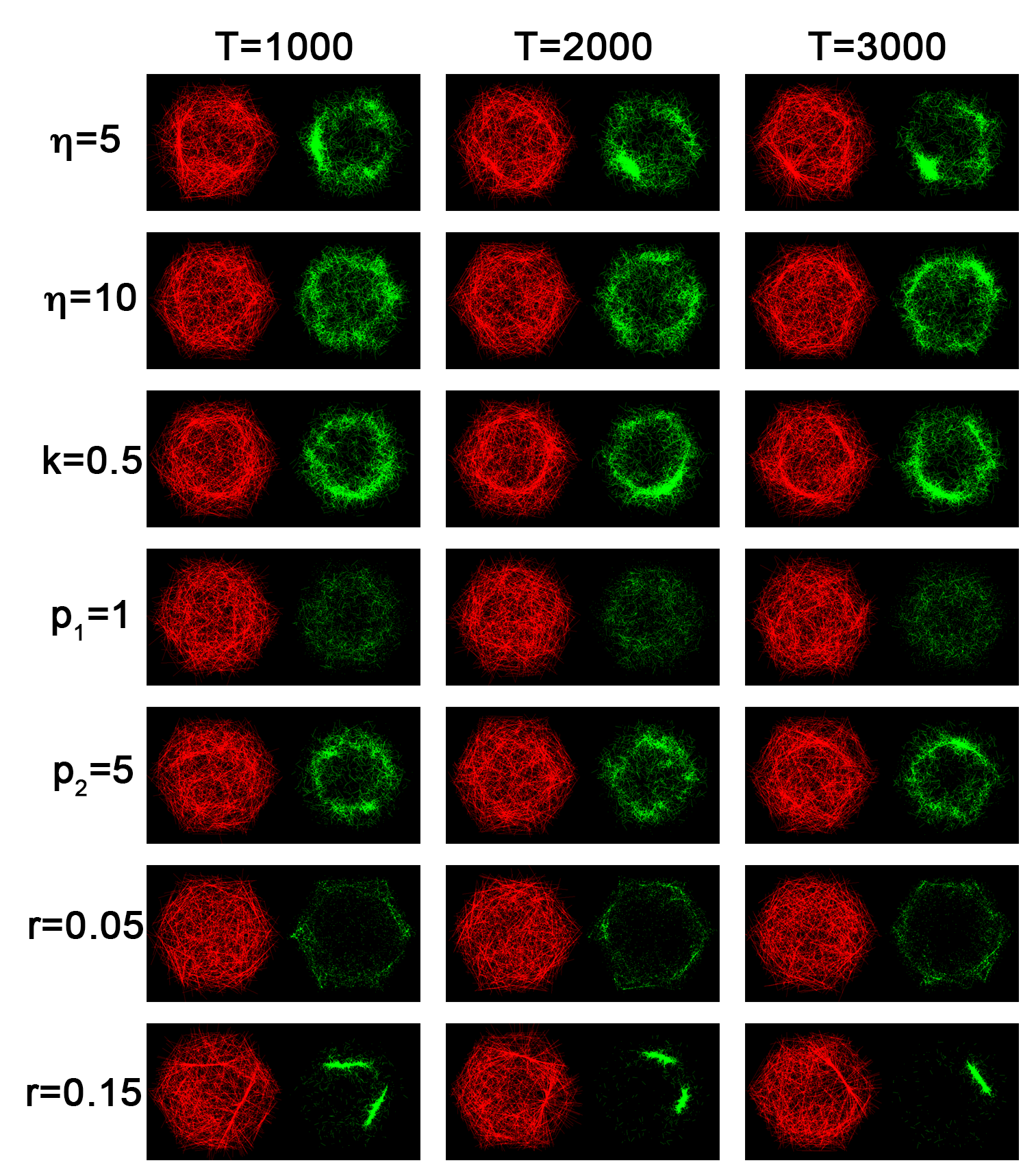

Supplement: S15 Fig — We identified cases where simulations did not end in a central aster from Figs 4 to 5 (marked with an *) and ran those simulations for longer times (T = 3000 time steps) to determine if those parameter sets merely delayed aster formation. We have noticed that two simulations ended in asters on the side (η = 5 and r = 0.15), two simulations have clumping on the periphery (p2 = 5 and k = 0.5), and three simulations don’t have any evidence of an aster (r = 0.05, p1 = 1, and η = 10). (TIF) [file pcbi.1006344.s022.tif]
